# Single unit action potentials in humans and the effect of seizure activity

**DOI:** 10.1093/brain/awv208

**Published:** 2015-07-17

**Authors:** Edward M. Merricks, Elliot H. Smith, Guy M. McKhann, Robert R. Goodman, Lisa M. Bateman, Ronald G. Emerson, Catherine A. Schevon, Andrew J. Trevelyan

**Affiliations:** 1 1 Institute of Neuroscience, Medical School, Framlington Place, Newcastle upon Tyne, NE2 4HH, UK; 2 2 Department of Neurological Surgery, Columbia University, New York, NY, USA; 3 3 Department of Neurosurgery, Icahn School of Medicine at Mount Sinai, New York, NY, USA; 4 4 Department of Neurology, Columbia University, New York, NY, USA; 5 5 Department of Neurology, Cornell University Medical Center, New York, NY, USA

**Keywords:** epilepsy, EEG, spike sorting, ictal core, ictal penumbra

## Abstract

Spike-sorting algorithms have been used to identify the firing patterns of isolated neurons (‘single units’) from implanted electrode recordings in patients undergoing assessment for epilepsy surgery, but we do not know their potential for providing helpful clinical information. It is important therefore to characterize both the stability of these recordings and also their context. A critical consideration is where the units are located with respect to the focus of the pathology. Recent analyses of neuronal spiking activity, recorded over extended spatial areas using microelectrode arrays, have demonstrated the importance of considering seizure activity in terms of two distinct spatial territories: the ictal core and penumbral territories. The pathological information in these two areas, however, is likely to be very different. We investigated, therefore, whether units could be followed reliably over prolonged periods of times in these two areas, including during seizure epochs. We isolated unit recordings from several hundred neurons from four patients undergoing video-telemetry monitoring for surgical evaluation of focal neocortical epilepsies. Unit stability could last in excess of 40 h, and across multiple seizures. A key finding was that in the penumbra, spike stereotypy was maintained even during the seizure. There was a net tendency towards increased penumbral firing during the seizure, although only a minority of units (10–20%) showed significant changes over the baseline period, and notably, these also included neurons showing significant reductions in firing. In contrast, within the ictal core territories, regions characterized by intense hypersynchronous multi-unit firing, our spike sorting algorithms failed as the units were incorporated into the seizure activity. No spike sorting was possible from that moment until the end of the seizure, but recovery of the spike shape was rapid following seizure termination: some units reappeared within tens of seconds of the end of the seizure, and over 80% reappeared within 3 min (τ_recov_ = 104 ± 22 s). The recovery of the mean firing rate was close to pre-ictal levels also within this time frame, suggesting that the more protracted post-ictal state cannot be explained by persistent cellular neurophysiological dysfunction in either the penumbral or the core territories. These studies lay the foundation for future investigations of how these recordings may inform clinical practice.

**See Kimchi and Cash (doi:10.1093/awv264) for a scientific commentary on this article.**

## Abstract (precis)

**See Kimchi and Cash (doi:10.1093/awv264) for a scientific commentary on this article**.

In patients undergoing surgical evaluation of focal neocortical epilepsies, Merricks *et al*. perform the first single-unit recordings of neurons in the ictal core and contrast their activity patterns with those of the penumbra. Single-unit spiking recovers rapidly after seizure termination, suggesting a network rather than cellular cause of post-ictal dysfunction.

## Graphical abstract

**Figure:**
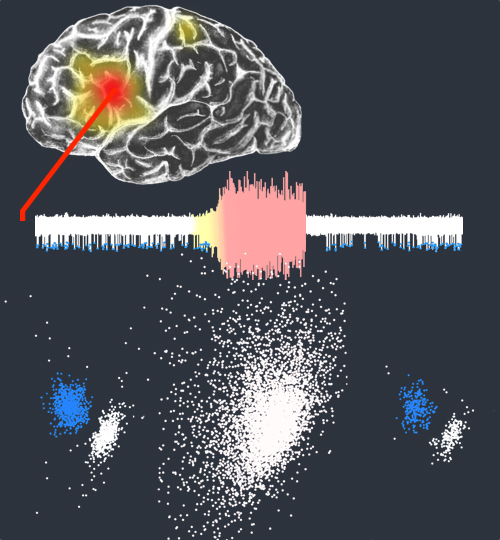
**See Kimchi and Cash (doi:10.1093/awv264) for a scientific commentary on this article**. In patients undergoing surgical evaluation of focal neocortical epilepsies, Merricks *et al*. perform the first single-unit recordings of neurons in the ictal core and contrast their activity patterns with those of the penumbra. Single-unit spiking recovers rapidly after seizure termination, suggesting a network rather than cellular cause of post-ictal dysfunction.

## Introduction

Action potentials are the means by which neurons send information rapidly throughout the brain. Analysis of neuronal firing patterns can therefore tell us a great deal about brain function, particularly for neurons close to the output of the motor system, or early in the sensory system (Hubel and Wiesel, 1977). It is also possible to scrutinize these action potential trains for clinically relevant information. For instance, in the context of epilepsy, rather than questioning how neuronal firing relates to the outside world, one might ask what the firing patterns tell us about the local brain state, and how they relate to the timing and frequency of both interictal events and seizures (Verzeano *et al.*, 1971; Ishijima, 1972; Babb and Crandall, 1976; Schmidt *et al.*, 1976; Babb *et al.*, 1987; Bower and Buckmaster, 2008).

Technological advances in microelectrode arrays (MEAs) mean that we can now gather data about many neurons simultaneously. These arrays were developed for use in brain–machine interfaces, and so the initial studies of their performance considered the long-term stability of recordings. Barrese *et al.* (2013)described a period of quiescence following array implantation in which isolatable single neurons gradually appear across the array over a period of weeks, and then disappear after months of recording. Another study examined the stability of action potentials recorded on microelectrode arrays, used to interface a computer with human primary motor cortex, over a period of 2 years (Suner *et al.*, 2005). There is little information, however, relating to the stability of unit isolation recorded using these MEAs in the acute/subacute time frame, as provided by their use in presurgical seizure monitoring.

Spike sorting analyses of extracellular recordings rely on units showing a stable action potential waveform, and so the occurrence of paroxysmal depolarizing shifts (PDSs) may confound such studies. PDSs are regarded as the intracellular correlate of epileptic discharges, and are characterized by reduced amplitude action potentials riding on the crest of the large depolarizations (Traub and Wong, 1982). However, PDSs have been described almost solely from animal studies, where intracellular recordings are feasible. Their occurrence in human epilepsy remains controversial, with several studies of human extracellular recordings failing to find any evidence of PDSs (Wyler *et al.*, 1982; Babb *et al.*, 1987; Stead *et al.*, 2010; Truccolo *et al.*, 2011). One explanation for these null findings relates to the location of the electrode placements.

We previously proposed the terms ‘ictal core territory’ and ‘ictal penumbra’ to distinguish between two qualitatively different activity patterns during seizures (Schevon *et al.*, 2012; Trevelyan and Schevon, 2013; Weiss *et al.*, 2015). These concepts arose from a large body of animal studies describing epileptiform activity in terms of localized, intense, hypersynchronous discharges (ictal core), with many neurons displaying PDSs (Kandel and Spencer, 1961*a*; Matsumoto and Marsan, 1964; Traub and Wong, 1982), associated with a marked inhibitory response in surrounding territories (penumbra) (Prince and Wilder, 1967; Dichter and Spencer, 1969*a*, *b*; Wong and Prince, 1990; Schwartz and Bonhoeffer, 2001; Timofeev *et al.*, 2002; Timofeev and Steriade, 2004; Suner *et al.*, 2005; Trevelyan *et al.*, 2006, 2007; Cammarota *et al.*, 2013), which appears to restrain the propagation of the focal pathophysiology. Note however, that even in the penumbra, there is typically increased firing over baseline activity levels (Schevon *et al.*, 2012). Recent studies of microelectrode array recordings in human subjects have now extended this description to include spontaneous (‘habitual’) human seizures. In both core and penumbral territories, one can find very large low frequency local field potentials recorded on subdural recordings from macroelectrodes on the surface of the brain, but of critical importance is that these recordings also allow the two territories to be distinguished by means of a pronounced phase-locked high gamma band signal visible only in the ictal core (Weiss *et al.*, 2013).

It is important to be clear about the distinction between the widely used term, the ‘seizure onset zone’ and the ictal core. The former is the best estimate of where a seizure begins, and is a fixed location reflecting what is happening at a single time point, namely the earliest time when seizure activity could be detected. In contrast the ictal core is a dynamic concept, describing which cortical territories are fully recruited at a given point in time, and so changes over the time course of the seizure.

We present here analyses of human single neuronal unit activity from both penumbral, and for the first time, core recruited territories. We asked if the ability to follow single units, as described recently by Truccolo *et al.* (2011), might depend on whether the MEA was located within the core or the penumbral territories at a given time point during a seizure. We investigated the stability of these units over time, how unit activity was altered during a seizure, and documented the recovery of unit activity after seizure termination.

## Materials and methods

### *In vitro* electrophysiology

All procedures were performed according to the requirements of the UK Animals (Scientific Procedures) Act 1986. Cell attached recordings were made in coronal brain slices prepared from juvenile (postnatal Days 14–20), wild-type, C57/Bl6 mice, as previously described (Trevelyan *et al.*, 2006, 2007). In short, animals were sacrificed by cervical dislocation, and 350-µm thick, coronal, brain slices were cut in ice cold artificial CSF containing (in mM): NaCl 125, KCl 3.5, NaHCO_3_ 21, MgCl_2_ 3, glucose 10. Slices were subsequently incubated at room temperature in artificial CSF with 2 mM CaCl_2_ and 1 mM MgCl_2_, before being transferred into a submerged recording chamber mounted on an upright Olympus BW51 microscope adapted for patch clamp electrophysiology (Scientifica micromanipulators and movable top plate). The bathing artificial CSF was heated to 32–36°C using a sleeve heater and heated stage (Warner Instruments). Epileptiform activity was induced by removing magnesium from the bathing medium (0 Mg^2+^ model). Patch clamp electrodes (4–7 MΩ) were filled with artificial CSF, to make cell-attached recordings from large, layer 5 pyramidal cells identified under direct visualization using differential interference microscopy. The seal resistance for these recordings always exceeded 1 GΩ.

### Human neocortical recordings

The human studies were conducted under the oversight of Columbia University’s Institutional Review Board and complied with all regulations. Adults with pharmacoresistant focal epilepsy undergoing chronic invasive EEG monitoring to identify the ictogenic zone prior to surgical resection, were implanted simultaneously with a 96 electrode, 4 × 4 mm MEA (Neuroport™ neural monitoring system, Blackrock Microsystems Inc.). Assessment of simultaneous video-EEG in the peri-ictal period was made by the clinical investigators (C.A.S., L.M.B.). The MEA was implanted into neocortical gyri, with the location selected based on presurgical estimations of ictogenic region, and so as to be included in the subsequent resection. Signals from the MEA were recorded continuously at 30 kHz per channel with 16-bit precision and a range of ± 8 mV, with a band pass filter from 0.3 Hz to 7.5 kHz. Further details of these recordings can be found in previous reports (Schevon *et al.*, 2008, 2009, 2012). Seven patients were implanted, but isolated habitual seizures were captured in only four: as we were particularly interested in the peri-seizure firing patterns, we restrict our analyses in this paper to those four subjects. All analyses were performed offline in the MATLAB (MathWorks) environment, using custom scripts and modified routines from the Chronux toolbox (http://chronux.org) (Mitra and Bokil, 2007). Box plots were created using online software (http://boxplot.tyerslab.com/).

### Single unit discrimination

For long-term monitoring of spike stability 180-s epochs were selected every 30 min for analysis of single unit activity, so as to allow automated handling of the large data sizes over prolonged time periods. For detailed analyses of the time period surrounding seizures, epochs were taken from 30 min prior to seizure onset, until 15 min post, data permitting. Long-term post-ictal recovery of units in the ictal core was assessed using the 2 h immediately following seizure termination, as a single epoch. Multi-unit activity was extracted from the MEA signals using a second order Butterworth filter with a band pass between 300 Hz and 3 kHz to remove slower, synaptic events from the signal. Extracellular action potentials were located using a negative threshold set by the background noise level (threshold = 5.92 × median of the absolute deviation of the signal; this approximates to four times the standard deviation of the signal, but the median was used instead because it is distorted less by outlying points due to action potentials, and so is more robust to differences in firing rates; Quiroga *et al.*, 2004). Waveforms from these locations were then stored as 1.6-ms epochs (0.6 ms prior, to 1 ms post-detection). Spikes were clustered automatically based on their first three principal components using a *k-*means algorithm robust to outliers. Spikes were at first over-clustered; nearby clusters were then joined if they showed weak separation according to a measure of their interface energy (Fee *et al.*, 1996). All clusters were then inspected manually, and only clusters displaying all of the following criteria were accepted: (i) clean separation from all other units and background activity in the projection onto Fisher’s linear discriminant; (ii) <1% contamination of the absolute refractory period in the interspike interval; and (iii) no clear outliers of waveform shape using a *χ*^2^ probability distribution (Hill *et al.*, 2011). For long term clustering of putative single units in core patients, the seizure epoch (Patients 1 and 2, 30 to 120 s) was blanked once loss of separable features during this time point was confirmed. This allowed accurate continuous sorting of units before and after the seizure. Principal components were calculated simultaneously for all waveforms from all epochs during long-term clustering, so as to confirm the cluster of spikes belong to the same unit as before (Supplementary Fig. 2).

### Electrode characteristics

Probability density histograms were calculated on the first two principal components of the unclustered multiunit spikes. One hundred bins in each direction were used for the 2D histograms, and smoothed with a moving average of five bins (Figs 2C and 5C). Within electrode, all epochs’ dimensions were normalized to the maximum and minimum of the first epoch in each dimension to avoid effects of extreme outliers in single epochs. 2D cross correlations of the resultant smoothed probability density histograms were calculated for both intra- and interelectrode comparisons, to calculate stability of features through time within electrode, and specificity of features recorded between electrodes (Fig. 6A and B). Coefficients from all correlations were found to be non-normal (Kolmogorov-Smirnov test for normality; maximal *P* < 0.0001), therefore significance was assessed with the Mann-Whitney U-test.

A possible problem with this cross-correlation analysis may arise from particularly high levels of background multiunit activity, which could give rise to a biased electrode signature. An example of this is shown in Fig. 2C: the imbalanced distribution, with the great excess of spikes in the maintained background activity, may give rise to an exaggeration of intra-electrode similarity, which does not necessarily reflect what we were trying to assess, which was the stability of individual units. To avoid this biasing effect, a secondary, subtractive method was devised (Fig. 6C, D and Supplementary Fig. 1). The first, baseline probability density histogram was subtracted from each subsequent histogram; as the number of background distal spikes was well maintained, such a subtractive method removes their influence. Similarly, a single unit showing no change in waveform or firing rate would result in near-zero values and so the subtractive method highlights only alterations in well-separated units’ firing patterns or waveform.

### Maintenance of single units pre- and post-seizure

Maintenance of single units was further confirmed by clustering equal duration pre- and post-ictal epochs (10 min each) independently. This was done to avoid the biasing effect of assigning post-ictal spikes to the most similar pre-ictal cluster that may occur through simultaneous clustering of both time periods. An assessment of drift in principal component space between units clustered independently in the two time points was calculated from the centroids of clusters (Fig. 7A). The drift coefficient corresponds to the distance between the two time points’ cluster centroids divided by the distance of the first time point’s centroid from the mean value in principal component space. To assess the significance of this drift coefficient, the true results were compared to the mean results of comparing clusters to non-equivalent clusters in the other time point at random over 10 000 iterations. Statistical comparisons were made by comparing each unit to a different unit, chosen at random from the population 10 000 times, and the mean result of these comparisons was taken as the single trial value for that unit. This strategy had the advantage of maintaining the same number of sampled units.

The maximal cross correlation value of the mean waveforms from the two epochs’ clusters (Fig. 7B) was used to confirm that multiple clusters corresponded to the same unit. As a high correlation coefficient would be expected due to all waveforms being selected for having a noticeable downwards deflection, these results were also compared to the mean results of comparing mean waveforms between non-equivalent clusters in the other time point at random, over 10 000 iterations, using the same method as described above. The delay in return of activity from cluster-able single units was assessed by clustering the 2 h immediately post-seizure termination, and the timing of the first waveform from each cluster was used as the delay.

Statistics are presented as mean ± standard error of the mean (SEM) unless otherwise specified. Changes in firing rate were determined to be significant when beyond 3 standard deviations (SD) of the cell specific Poisson distribution, as estimated as the square root of the firing rate divided by epoch duration (Supplementary Fig. 3; Vajda *et al.*, 2008).

## Results

### Intense firing is associated with changes in extracellular spike shape

The PDS is regarded as the intracellular hallmark of an epileptic discharge. There is a strong presumption that these changes should also be apparent in extracellular recordings, but to check this explicitly, we made cell attached recordings from visually identified, layer 5, pyramidal neurons in brain slices prepared from young mice, during epileptiform events induced by removing Mg^2+^ ions from the bathing medium (0 Mg^2+^ model; Supplementary Fig. 4). Cell attached recordings isolate the membrane currents of the patched cells without interference from activity in neighbouring neurons. These recordings showed that during the seizure-like event, there occurs a large decrease in the amplitude of the action potential currents, with an associated broadening of the spike (Supplementary Fig. 4C). The scatter plots of the amplitude of the action potential current versus the interspike intervals showed several notable features (Supplementary Fig. 4D). First, there was a clear evidence for a refractory period in all cells, with the shortest interspike intervals being followed by the smallest action potentials. Exponential fits to the amplitude versus interspike interval plots gave a time constant, τ = 108.9 ± 59.6 ms (*n* = 5 cells from five animals). Second, by identifying the smallest amplitude action potential current during the baseline period, and setting a threshold just below that, one could separate two groups of action potentials: those occurring during baseline (large) and those occurring during the seizure (small). The most intense firing rates (smallest interspike intervals) occurred during ictal events, but the wide and overlapping range of interspike intervals for both the small and large action potentials showed the seizure activity was associated with a greatly extended refractory period relative to the baseline activity. The likely cause is the huge level of synaptic drive during these *in vitro* epileptiform events. Large synaptic currents are also apparent during human seizures *in vivo*, and so warrant a re-examination of the human spike sorting data.

### Stable unit identification in human MEA *in vivo* recordings

**Figure 1:**
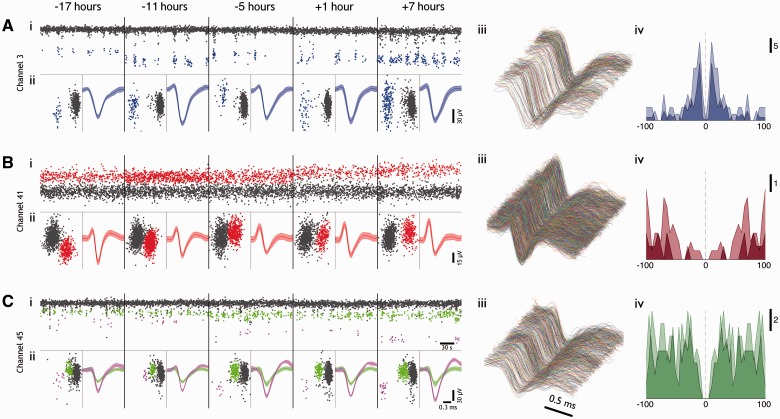
**Stability of single unit features over 24 h in humans.** (**A–C**) Typical features from three channels in Patient 1, from 3-min epochs every 6 h, over 24 h, during which time there occurred a seizure (between the 6- and 12-h time periods). Times represent time relative to seizure onset. For each channel we show (**i**) first principal component versus time; unit showing clean separation from the background noise of more distal units (grey), highlighted in colour (blue, red, green and pink). (**ii**) *Left*: Full epoch’s first versus second principal component, and *right*: mean waveform (± 2 SD) of all spikes in cluster. (**iii**) Every waveform from each epoch plotted contiguously (only the ‘green’ unit in **C** is depicted). Note evidence for stability of both a bursting cell, in **A**, and for a highly active cell, in **B**. While there is a drastic change in mean waveform over 24 h in **B**, the maintained distinct cluster in **B**(**ii**) without the presence of other well separated activity imply strongly that this is a cell showing drift relative to the electrode tip. (**iv**) Autocorrelograms from time points −5 and +7 h relative to seizure from the same cells as (**iii**) (light and dark, respectively), over ± 100 ms, using 5-ms bins. Note the maintained intrinsic firing properties within each cell, and different properties between cells.

We therefore performed spike-sorting analyses to identify putative single neuronal units from 96 channel MEA recordings in four patients with intractable focal neocortical epilepsy. We were able to isolate 305 putative single units successfully from a subset of channels in all patients (Supplementary Table 1), using a standard negative voltage thresholding technique and clustering of principal component features [false positive estimate from 2 ms refractory period violations: 0.204 ± 0.769% (mean ± 2 SD); false negative estimate from Gaussian distribution of detected thresholds: 0.003 ± 0.059% (mean ± 2 SD); as per Hill *et al.*, 2011]. The waveform shapes of these extracellular action potentials were stable during interictal epochs over at least 24-h periods in many cells, including from periods either side of a seizure (Fig. 1). In the example shown in Supplementary Fig. 2, the same unit is followed across two seizures, 16 h apart.

To illustrate the analysis, we present in detail the data for one such 24-h period in Patient 1, where we analysed brief (180 s) epochs every 6 h. For these initial analyses, we excluded epochs when there was seizure activity. We identified 76 single units in total, and of these, 54 units (71%) maintained their waveform characteristics throughout the non-seizure epochs during this 24-h period, four units (5%) were lost and did not return, and 12 (16%) were not visible during the first analysed epoch, but appeared and persisted in subsequent epochs. There was no particular association of the appearance of new units with seizures. One unit was visible only in a single epoch, and five units (6.5%) were lost temporarily, for one or more epochs, but became evident again by the end of the 24 h. In cases where the unit waveform altered over a 6-h period (e.g. Fig. 1B; note, however, that the cluster separation was well maintained), we reanalysed more frequent time points (180-s epochs every 30 min) to follow the changes in principal component space over time more closely (Supplementary Fig. 2). We noted instances where one unit changed while others remained stable (Fig. 1C). Notably, the firing patterns shown by individual units provided further confirmation of the stability of these recordings: units displayed a range of firing features, including intermittent bursting (Fig. 1A) and fast spiking patterns (Fig. 1B), these identifying firing patterns also persisted over time [Fig. 1A–C(iv)].

**Figure 2:**
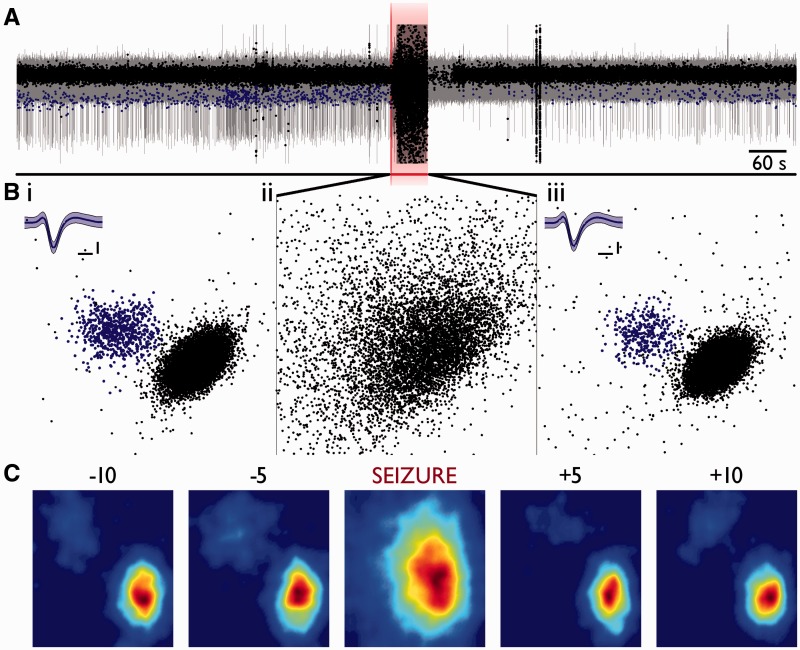
**Loss of unit-specific features during seizure in core, recruited territories.** (**A**) Example trace from one channel in Patient 1 (recruited territory) for 10 min prior to seizure onset, to 10 min post-seizure termination. The first principal component values from detected spikes have been overlaid; the blue data points all represent spikes from a single, cleanly separated unit. Note the stability of the principal components up until seizure onset whereupon all discernable features are lost. The same unit as in the preceding 10 min can also be seen to make a recovery in the following 10 min, albeit with a much diminished firing rate. (**B**) First two principal components are plotted against each other. They show a clearly separable unit in blue and, *inset*, mean waveform (± 2 SD) from: (**i**) 10 min prior to seizure; (**ii**) during seizure (60 s total epoch); and (**iii**) 10 min following seizure termination. Note the maintained waveform and corresponding principal component values in **iii** relative to **i**, despite no evidence for the same unit during the seizure in **ii**. Both axes are maintained throughout, and principal components were calculated simultaneously. Waveform inset scale = 0.2 ms, 20 μV. (**C**) Kernel density estimate histograms (sigma = 5, 100 bins) of principal components 1 (abscissa) and 2 (ordinate), from 60-s epochs from 10 and 5 min prior to seizure onset, during the seizure, and 5 and 10 min post-seizure termination, normalized to the dimensions found in the 10 min prior epoch. Red denotes the highest probability of finding a spike, blue the lowest.

The plot of the first two principal components for the collected units from a given electrode has a distinctive distribution, a kind of electrophysiological signature. Because many such signatures were being collected simultaneously from multiple electrodes, we can use this between-electrode variance to assess the temporal stability of the recordings. The between-electrode comparisons are by definition comparisons of different units, and so provide us with an estimate of the variance of the electrophysiological signatures. To do this, we smoothed the probability density histograms (normalized in feature space, Figs 2C and 5C) of 60-s epochs from 10 min prior to seizure onset, to 5 min post, relative to a baseline 60-s epoch from 30 min prior to seizure onset, and performed cross-correlations on these smoothed plots (Fig. 6A and B). These cross correlations were performed on all channels that showed evidence of activity distinct from the homogeneous background multi-unit activity (Patients 1–4: *n* = 35, 49, 30, and 32 channels, respectively). Cross correlations were performed between all electrodes within each epoch, for each patient separately (Fig. 6A and B). This demonstrated a low similarity of different electrode recordings seen during baseline periods, a result that was highly reproducible between patients (global mean ± 2 SD correlation coefficient from non-seizure epochs for all patients: 0.14 ± 0.01), and which represents a large spread of possible electrode signatures. In contrast, the within electrode, temporal analyses showed extremely high correlations for all non-seizure time points, relative to the first epoch at 30 min prior to seizure, and again was highly reproducible between patients (global mean ± 2 SD correlation coefficient from non-seizure epochs for all patients: 0.97 ± 0.004).

### Spike sorting differences between the penumbral and ictal core territories

We next analysed the stability of unit spike shape around the times of seizures. The positioning of the MEAs was made on the available clinical evidence about the best estimate of the location of the seizure onset zone. Subsequent analyses showed that in two patients, this territory was fully incorporated into the core recruited territory during seizures, while in the other two patients the MEA was judged to have been placed in the penumbral territory (Schevon *et al.*, 2012). This important point is worth restating: that even within the clinically defined seizure onset zone, some territories were not fully recruited to the ictal core territory, illustrating the difficulties of defining the seizure onset zone precisely. We made use of this chance distinction between the recording locations, to determine the stability of the unit recordings over time, with particular interest in whether units could be followed during seizures in the penumbral and the ictal core territories.

**Figure 3:**
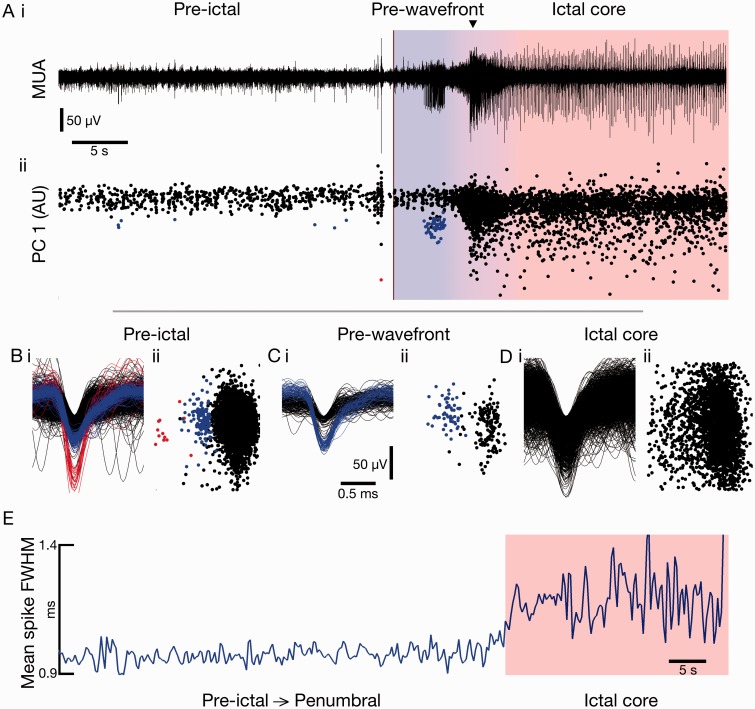
**Maintained waveform during propagation prior to local ictal onset.** Cortical regions that are later incorporated into the ictal core show maintained waveforms after seizure onset while they are still in the penumbra (prior to the wavefront reaching and incorporating that territory). [**A**(**i**)] Example MUA (300 to 3000 Hz) trace from Patient 2 and (**ii**) accompanying first principal component through time of detected spikes, from 30 s prior to global seizure onset (dark red line) until 30 s after. For the first 4.8 s after seizure onset the territory local to the electrode remains penumbral, prior to the wavefront reaching the region (blue shaded area), after which the territory becomes incorporated into the ictal core (red shaded area). Note the maintained spike height and first principal component while in the penumbra, and loss of specificity once incorporated into the ictal core. (**B–D**) (**i**) Waveforms and (**ii**) their first two principal components from the channel shown in **A**, from 5 min pre-ictal, the penumbral 4.8 s, and the ictal core, respectively. Note the maintained waveform and associated features of the blue unit during the penumbral period (**C**) followed by obscurement when incorporated into the ictal core (**D**). (**E**) Mean spike full-width at half-maximum (FWHM) of all single units from Patient 2, aligned in time to local incorporation into seizure (ictal core shown in red shaded area), showing that even when including spikes from the same principal component region despite a lack of defined clusters, the spike width within clusters increases at local onset, and not while in the penumbral region.

**Figure 4:**
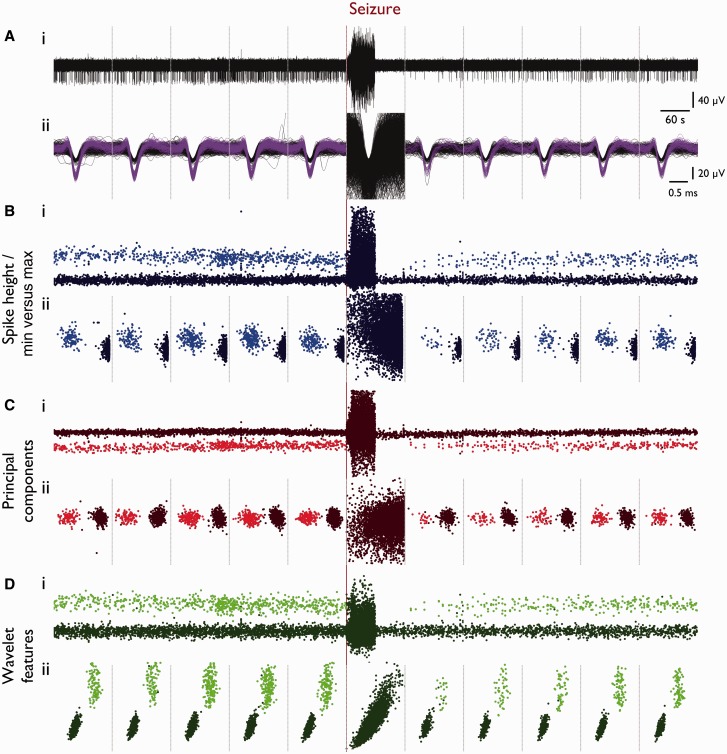
**Loss of specificity in core territory in all cluster-able features.** [**A**(**i**)] Example trace from one channel in Patient 1 (recruited territory) from 10 min prior to seizure onset until 12 min post-seizure onset, segmented into 2-min epochs (grey dotted lines, used throughout). (**ii**) All waveforms from each 2-min epoch shown in **i**, with background noise in black and single unit in purple. (**B–D**) Multiple cluster-able features, with single unit as lighter colour throughout. [**B**(**i**)] Spike height plotted versus spike time and (**ii**) spike minimum versus maximum for each epoch. [**C**(**i**)] First principal component versus time and (**ii**) first versus second principal component for each epoch. [**D**(**i**)] First wavelet feature versus time; and (**ii**) first versus second wavelet feature for each epoch. Note loss of features throughout during seizure epoch.

In recordings from MEAs that eventually were fully incorporated into the ictal core territories, there were also preceding periods of penumbral activity, as defined by the large amplitude low frequency signal with low levels of spiking. Notably, for these ‘penumbral periods’, spike shapes were preserved in all electrodes (Fig. 3). At the arrival of the ictal wavefront, however, and throughout the seizure subsequently, many action potential waveform characteristics change, including the principal components, wavelet features, waveform maxima and minima, and the non-linear energy (Figs 2–4). In short, all features on which spike sorting might be performed are altered, preventing any useful strategy for following a single neuron’s activity patterns through the seizure.

After recruitment there was a notable increase in the number of large amplitude first principal components (Fig. 2B), suggestive of superimposed spikes caused by hypersynchrony, and reflecting previously observed synchronized bursting across all electrodes in the 4 × 4 mm MEA at this time (Weiss *et al.*, 2013). When these features are plotted out in a phase plot, the clusters that define a single unit get lost inside a great cloud of data points. We therefore considered two alternative hypotheses to explain the inability to cluster. First is the occlusion hypothesis, which is that units preserved their shape, but clustering was prevented by the appearance of other units. The second is the PDS hypothesis, which is that spikes change their shape. To distinguish between these two hypotheses, we plotted the change in spike half width for units throughout the seizure, from within the same small area of the principal component phase plots as the original cluster (Fig. 3E). This showed an increase in spike half width at the time of recruitment for data points within that area of the phase plots, thus indicating that the occlusion hypothesis alone cannot account for all observations, but remaining consistent with the PDS hypothesis.

The within electrode temporal correlations were high for all interictal epoch comparisons (referenced to a time point 30 min before the seizure; Fig. 6A and B). Within the core territory (Fig. 6A), this correlation dropped significantly during the seizure compared with the baseline reference epoch (Patient 1, Seizure 1: 47% drop from baseline, *P* << 0.001, *n* = 35; Patient 2, Seizure 1: 10.4% drop, *P* << 0.001, *n* = 49; Mann-Whitney U-test). There were instances during interictal periods when the temporal correlations dropped significantly, but this was always a far smaller change than shown during the seizure event (Patient 1, maximal interictal shift = 1.3%, *P* = 0.02; Patient 2, maximal interictal shift = 0.8%, non-significant). The ictal core activity was also characterized by a significant rise in the between-electrode correlations (Fig. 6A; comparison of seizure between-electrode distribution with a time epoch 10 min prior; Patient 1: *P* << 0.001, Patient 2: *P* = 0.003, Mann-Whitney U-test), which occurred because the electrode signatures lost their most distinguishing features (i.e. the discrete clusters which represent single units).

**Figure 5:**
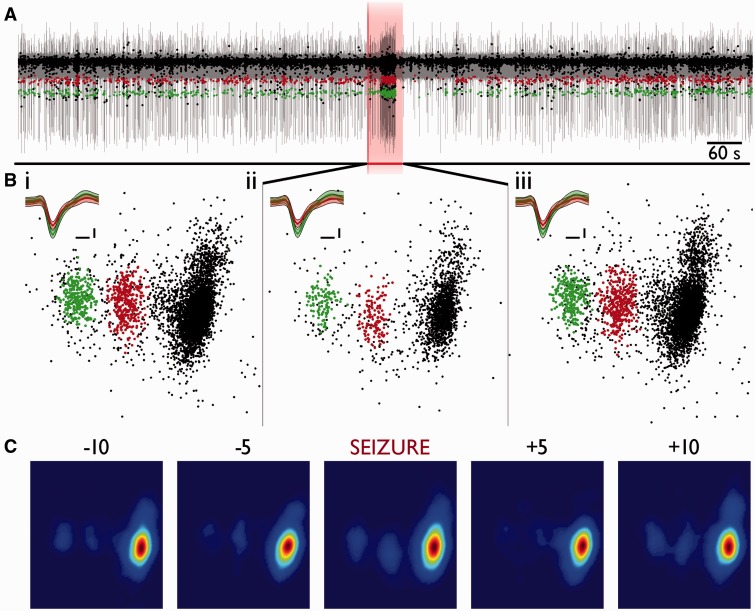
**Maintained waveform and features during seizure in penumbra.** Same format as for Fig. 2, showing the preserved electrophysiological signatures recorded within the penumbral, non-recruited territory [Patient 3 (shown in Figure 7g, Seizure 1 in Schevon *et al.* 2012)]. The large amplitude, low frequency signals indicative of seizure activity, corresponded approximately to the period of increased unit activity. Note that the pink shaded territory indicates the sampling period for **B**(**ii**) [60 s; the same duration as in Fig. 2B(ii)], but that in this figure it also includes short pre- and post-ictal periods. (**A**) The 300 Hz to 3 kHz bandpass filtered trace from a single electrode, with two well separated units indicated in green and red. (**B**) The distributions of the first two principal components for these units, together with many other units which are shown in black. All scales are maintained through, inset scale = 0.2 ms, 20 μV. (**C**) The Kernel density estimate histograms for the distributions shown in **B**. Note how the two units are clearly separable during each time point, and that the principal components (and Kernel density estimates) are stable, even though there is a marked rise in firing frequency during the seizure relative to periods both preceding and following the seizure. The subdural EEG from this seizure is shown in Schevon *et al.* (2012; Figure 7g, Seizure 1).

**Figure 6:**
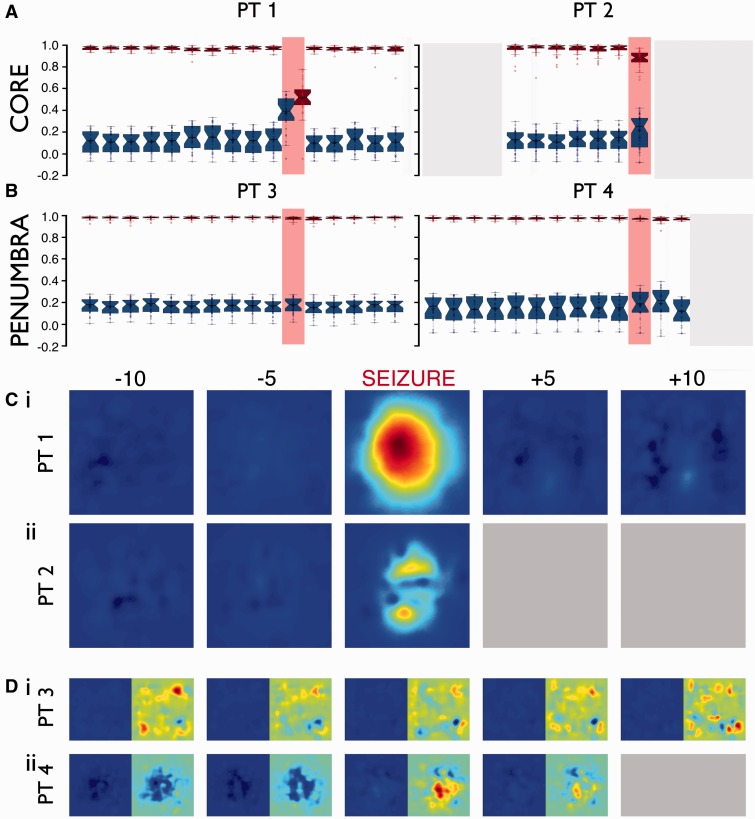
**Loss of separable features from single units is characteristic of core recordings, not present in penumbra.** (**A** and **B**) Cross correlations of Kernel density estimate histograms as shown in Figs 3C and 5C, for 60-s epochs from 10 min prior to seizure onset to 5 min post termination in all four patients [core in **A**: Patient 1 (30 electrodes), Patient 2 (45); penumbra in **B**: Patient 3 (30), Patient 4 (22)]. Red boxplots show intra-electrode cross correlation coefficients relative to the Kernel density estimate histogram 30 min prior to onset. Blue boxplots show interelectrode average cross correlation coefficients relative to each other electrode during that epoch. The strong correlations over time within each electrode during baseline periods compared to the weak correlations between electrodes show that these features are characteristic of activity specific to each electrode. During the seizure, in core recordings (**A**), the intra-electrode correlation is lost due to breakdown of features and the similarity between electrodes is raised due to similar loss of specificity, as seen in Fig. 3B(ii), in all electrodes. Recordings from penumbral territories (**B**) show no such alteration during seizure with continued specificity within electrodes. Strong correlations within electrode plausibly arise due to well-maintained background noise of more distal cells’ spikes. Grey areas denote missing data. (**C** and **D**) The mean results over all electrodes of a subtractive method whereby the first Kernel density estimate histogram was subtracted from the others. The background noise of more distal cells’ spikes is maintained thereby removing it during subtraction, leaving only alterations in unit specific activity. Resultant mean subtractions from core patients are shown in **C**, colour normalized to maximal value in Patient 1, showing a larger alteration during seizure relative to other time points. Penumbral recordings show no such alteration during seizure as shown in **D**, with values normalized to the colour axis in **C** on the *left*, and normalized within patient on the *right*.

In contrast, in penumbral territories, action potential waveform shape and associated features were maintained throughout the seizure for the majority of units, accompanied by a preservation of features in principal component space (95% and 76% unit retention during seizures, for Patients 3 and 4, respectively, relative to the pre-ictal period) (Supplementary Table 1). There was no significant difference in the cross-correlation analyses for any time point in the penumbral recordings. This result indicates that in penumbral cortical areas, despite clear evidence for the seizures directly influencing firing rate (Fig. 5A), cells did not undergo paroxysmal depolarizing shifts, nor was there sufficient superposition of spiking to distort summated waveforms. Because the unit clusters persisted, the between-electrode correlations also remained very low during the seizures in the penumbra (Fig. 6B). This difference between penumbral and ictal core activity was confirmed by a second analytical method based on subtracting the baseline electrode fingerprint from subsequent time epochs, to identify times when there occurred large deviations in the signature (Fig. 6C and D).

### Peri-seizure firing patterns of units in the ictal core, and the post-ictal recovery of firing

**Figure 7:**
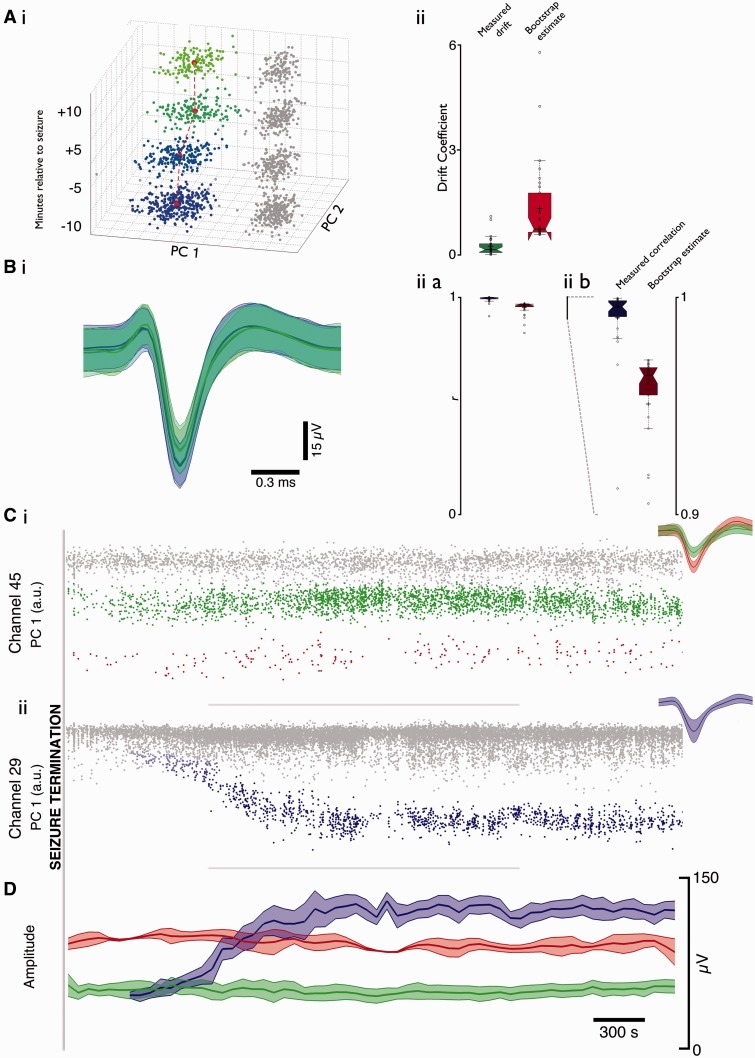
**Consistent cells evident both pre and post seizure in core recordings, with varied patterns of recovery.** The ability to record from the same cells after a seizure as beforehand discounts movement of the MEA, due to either patient movement or vasculature response, as the cause for loss of feature specificity during seizure. [**A**(**i**)] Example drift of cluster centres of a well isolated unit either side of a seizure (blue to green shows −10, −5, +5, +10 min relative to seizure onset, centre of cluster marked in red). (**ii**) Drift coefficient of mean post-seizure centre relative to mean pre-seizure centre (calculated in 3D space; 2D shown in **i**), from each cleanly separated unit with activity in both time points. Coefficient was calculated as the distance moved, divided by the prior time point’s distance from zero in principal component space. *Left* (green) shows actual drift coefficient; *right* (red) shows drift coefficient as determined by comparing each unit pre to a random different unit post 10 000 times. [**B**(**i**)] Mean waveforms (± 2 SD) from time points shown in **A**(**i**), colours maintained. [**B**(**ii**)] Cross correlation coefficients of mean waveform prior to seizure against mean waveform post seizure. (**a**) Left, resultant correlation coefficients, and right, correlation coefficients from comparing each unit prior to a random different unit post, 10 000 times. (**b**) Spikes were detected with a negative threshold, so all mean waveforms would be anticipated to show a highly correlated shape. Expanded view of (**ii**)**a** shows significant difference between waves found pre and post relative to arbitrarily compared waves. (**C**) First principal component over time for two example channels over a 2-h post-ictal period from Patient 1. Well isolated units are highlighted in green and red in channel 45 with unclassified units in grey. Note the immediate return of waveform after the seizure of both units. Channel 29 shows the simultaneous return of a unit with a drift in amplitude after the seizure, fading from grey to blue once distinct from the background activity. Insets show mean waveforms ± 2 SD for these clusters. (**D**) Mean (± 2 SD) amplitude for the three units shown in **C**, over the same time period. Note the stability in the red and green units at the same time as a sizeable alteration in amplitude of the blue unit.

We next examined the recovery of single units after being incorporated into the ictal core territory in the individual patient for whom we had both pre- and post-ictal recordings (the recording in Patient 2 was disrupted towards the end of the first seizure recorded, thus preventing any analysis of the post-ictal period). We performed this analysis on two equivalent duration (10 min) epochs immediately before and after the seizure, clustered independently to avoid biasing the results due to automatically matching pre- and post-ictal units as the same cell. We identified 40 cells using these shorter duration epochs, of which 30 were present both before and after the seizure, six cells were only present in the pre-ictal period (‘lost’ during the seizure), and four were present only in the post-ictal period. We further analysed the 30 persistent units by deriving a coefficient of drift of the centroids of the clusters in 3D principal component space. The coefficient displayed a significant similarity when compared to the mean values obtained from comparing the relative drifts of each unit to other units at random for 10 000 trials [mean ± 2 SD, observed: 0.26 ± 0.52; mean results from shuffled data (10 000 trials): 1.33 ± 2.35, *P* << 0.001, Mann-Whitney U-test] (Fig. 7A). This shows that the waveforms found in the post-ictal period were significantly more similar in shape to their corresponding pre-ictal waveform than would be expected by chance. Further confirmation in the preservation of recordings from the same cells was found in the cross correlation of mean waveforms from each cluster, between the pre- and post-ictal epochs. While a high correlation coefficient would be anticipated due to all waveforms being pre-selected based on their large amplitude negative deflections, the distribution of coefficients found was significantly different from the distribution of mean coefficients found from 10 000 trials of comparing each pre-ictal waveform at random to the post-ictal waveform of a confirmed different unit (mean ± 2 SD, real: 0.99 ± 0.03; 10 000 trials: 0.95 ± 0.06, *P* << 0.001, Mann-Whitney U-test) (Fig. 7B).

**Figure 8:**
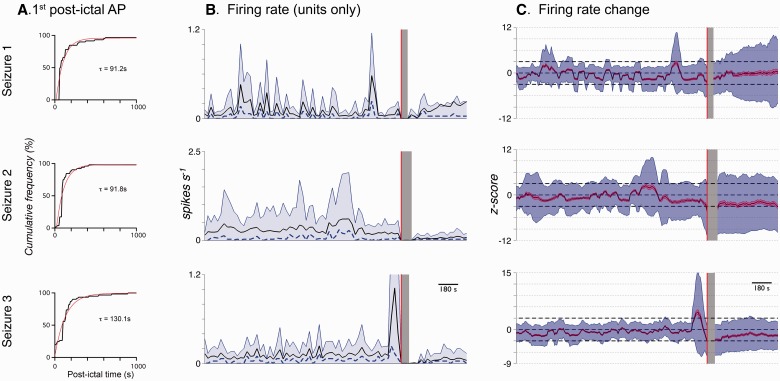
**Post-ictal recovery of population firing properties.** Firing properties for Seizures 1 to 3 in Patient 1 (recruited territory). Red line denotes seizure onset and grey area shows the seizure, which was blanked out for spike sorting purposes, used throughout. (**A**) Cumulative frequency plots for the time of first post-ictal action potential within clusters (black) and exponential fits to each (red). (**B**) Mean firing rate (black), median firing rate (dashed blue) and 90th percentile of firing rate (shaded blue region). (**C**) Z-scored changes in firing rate from each cell calculated in 60-s bins every 10 s, relative to the cell’s firing rate during the pre-ictal 30 min. Zero therefore means no change in firing rate, with positive values being an increase, and negative values a decrease. Dashed black lines at ± 3 denote significance levels used for changes in firing rate (Supplementary Fig. 3). Mean alteration is shown in black, mean ± SEM. is shaded red, and mean ± 2 SD is shaded blue.

The period of post-ictal quiescence varied widely between units, so to capture this, we extended these analyses up to 2 h post-seizure (Seizure 1, 59 units; Seizure 2, 52 units; Seizure 3, 61 units). Units predominantly reappeared without any evidence for change in waveform, or position in principal component space [Fig. 7C(i)]; however, 3 of 52 units from Seizure 2 and 3 of 61 units from Seizure 3 (one equivalent unit across both seizures) showed a visible slow return over many minutes of waveform amplitude and associated position in principal component space, despite the simultaneous presence of stable units [Fig. 7C(ii) and D]. Classifiable single units on average showed a prolonged quiescence after the final seizure discharge before the first post-ictal action potential [Fig. 8A; Seizure 1: 123 ± 17 s, 57 of 59 units (two did not return) from 35 channels; Seizure 2: 117 ± 11 s, 51 of 52 units (one did not return) from 33 channels; Seizure 3: 157 ± 19 s, 61 of 61 units, from 41 channels], albeit with a large variance shown within this data set (Seizure 1, 56–621 s; Seizure 2, 10–422 s; Seizure 3, 15–898 s). The cumulative histograms of the time to first action potentials for all units for the three seizures were quite stereotyped. Single exponential fits to these gave an average τ_recovery_ = 104 ± 22 s (mean ± SD).

Finally, we examined fluctuations in the mean firing rate across all single units before and after recruitment to the core ictal territory (Fig. 8B and C). There was marked fluctuation with time in the 30 min before the seizure, and from this we estimated the variance of activity in this population of identified single units, with reference to the mean firing rate over that entire 30-min period. Following each seizure, there was a gradual recovery of the population firing rate, returning to within 3 SD of the preictal rate within 100 s in all three seizures.

## Discussion

Our analyses confirm previous studies of MEA recordings in humans (Hochberg *et al.*, 2006; Truccolo *et al.*, 2011) and animals (Suner *et al.*, 2005; Gilja *et al.*, 2006; Linderman *et al.*, 2006) that these devices can isolate single unit neuronal firing patterns. These latter studies, in which the long-term stability of the units was explicitly addressed, were of chronic implants, but seizure monitoring is necessarily in the acute/subacute timeframe (days to weeks). These recordings demonstrate that persistently stable units can be found within this time frame too. More importantly, we highlight substantial changes in recording stability during seizures in particular cortical territories (ictal core), while other cortical areas (penumbra) show stable spike shapes, as has been noted by others (Truccolo *et al.*, 2011).

The ictal core is defined by local hypersynchronous discharges as indicated by the incidence of much larger ‘units’ starting at the time of the ictal wavefront, and also hypersynchrony over ranges of several millimetres (Weiss *et al.* 2013). There is also evidence of PDSs in many neurons, as indicated by distortion of any clearly separable clusters, although the evidence is less direct, and by analogy with comparable events in non-human recordings. The best direct evidence would come from intracellular recordings, which cannot be justified on clinical grounds.

The ictal penumbra, on the other hand, is characterized by a small increase in activity averaged across all neurons, but with a large amount of variance, with some neurons actually showing marked decreases in their firing rates. Such activity is seen immediately before the invasion of the ictal wavefront, and also in other MEAs that are never incorporated into the ictal core territories. There is no evidence of PDSs in the penumbra, and the activity, while clearly being influenced by the core activity elsewhere, cannot be said to be hypersynchronous. Notably, this penumbral pattern of activity appears to correspond precisely with that reported in other studies of human multi- and single units during seizures, with non-hypersynchronous activity and the ability to spike sort throughout the duration of the seizure where attempted (Truccolo *et al.*, 2011; Bower *et al.*, 2012), and so we contend that these other unit recordings are penumbral. In short, these studies appear to report recordings from units that were not recruited into the ictal core. Although the ictal core and penumbral territories differ in the pattern of activity, both are likely to experience compromised network function, albeit compromised in different ways (Trevelyan *et al.*, 2013).

The penetrative MEAs that allow for single unit studies, sample from a small area of cortex (4 × 4 mm). In addition, the sample size of high resolution recordings is also small: we only have two patient recordings that showed full ictal core activity, although notably, all four showed periods of penumbral activity. For these reasons, the context of these recordings needs to be provided by subdural electrocorticography, which has lower temporal and spatial resolution, but more extensive coverage, and critically, is used in many neurosurgical centres worldwide. For these reasons, subdural EEG recordings probably provide the best means of testing our hypothesized distinction of seizure activity into core and penumbral territories. To this end, we showed previously that the onset of a phase-locked, high gamma band EEG signal corresponded closely to the time when the arrays showed a transition into the fully recruited, ictal core pattern of firing (Weiss *et al.*, 2013). Using these biomarkers, we reanalysed contemporaneous subdural EEG recordings of old surgical resection cases to show that this new approach does indeed provide a coherent interpretation of the spread of neocortical seizures (Weiss *et al.*, 2015). With respect to the current study, the penumbral activity recorded by the MEAs (Patients 3 and 4 in this study) was concurrent with subdural electrode signals from elsewhere in the cortex showing evidence of ictal core activity (Schevon *et al.*, 2012; Weiss *et al.*, 2013). Finally, there are also clear parallels with a far more extensive animal data set, and so, altogether, this diverse data set indicates a consistent association of particular characteristic features defining the ictal core and the ictal penumbral territories.

To what cause, then, can we attribute the failure of spike-sorting algorithms in the ictal core? There are a number of possibilities, which we will discuss in turn: (i) that there is movement artefact, altering the relative positioning of the cell and electrode that gives rise to the unique waveform recorded; (ii) cell death; (iii) hypersynchrony results in interference between unit waveforms; and (iv) that cells experience PDSs with a consequent reduction in amplitude of the action potential related currents. Movement artefacts are certainly a possibility during a seizure, although it was noteworthy from the simultaneous video evidence that in both the ‘ictal core-recordings’, the loss of units occurred prior to any marked physical movements associated with the seizure.

There may also be microscopic movements of cells relative to electrodes, perhaps secondary to vascular muscle contraction or relaxation, associated with the intense neuronal activation. On the other hand, the near perfect recovery of spike shape for the great majority of units after the seizure is argument against this, because if there were significant movement, followed by recovery of the original position, this would be accompanied by local trauma, which even if small, would be expected to change the ‘electrical geometry’ from which the unique spike shape derives. What is certain is that movement artefact is not the sole cause of the loss of units during the seizure, because in that case, the loss of particular units would be accompanied by the gain of others. Thus, there must also be contributions from the other three explanations.

Cell death cannot explain the transient loss of units, which are recovered post-ictally, but cannot be discounted as the explanation for the small numbers of unit waveforms that disappeared persistently at the time of seizures. However, we also recorded features from new cells, suggesting that array movement (minor movement, most likely), rather than cell death is the cause of these persistent changes.

Hypersynchronous bursting at the microscopic scale is readily apparent from Ca^2+^ network imaging of rodent brain slices (Trevelyan *et al.*, 2006, 2007; Schevon *et al.*, 2012), and sampling considerations, in which single unit recordings invariably show intense bursting activity during seizures (Kandel and Spencer, 1961*b*; Matsumoto and Marsan, 1964; Ayala *et al.*, 1973), have also long argued for the same conclusion. Hypersynchrony is also apparent macroscopically in humans, as the spatial patterns of action potential firing across the MEAs show tightly synchronized discharges across several millimetres (Schevon *et al.*, 2012). This macroscopic hypersynchrony makes it likely that at the microscopic scale too, at single electrodes, most if not all neurons are firing. The increased dispersion of data points in the principal component plots (Fig. 3B) can only be explained by there being constructive interference between units, creating unusually large spikes. Thus we can conclude that hypersynchrony does indeed contribute to the failure of the spike sorting.

The contribution of PDSs, on the other hand is less clear. Our extracellular recordings are certainly consistent with the model proposed by Traub and Wong (1982), which indicated the prominent role of sustained voltage-gated conductances such as NMDA and voltage-gated Ca^2+^ channels in shaping the PDS. These channels certainly exist in human neurons, and there is evidence for the requisite synaptic bombardment to trigger plateau potentials during human seizures. The definitive proof of PDSs, however, would require intracellular recordings to be made, and this is not feasible for human recordings.

In fact, there are several reasons for expecting spike shape to alter. It may happen secondary to changes in Na^+^ and K^+^ distribution inside and outside cells, or by changes in cell volume or the extracellular volume. These changes may have been expected to be associated also with DC shifts in the extracellular recordings (Gumnit and Takahashi, 1965; Ikeda *et al.*, 1996), but we only observed such a shift in a single instance. Patch clamp demonstrations of PDSs are also relevant to this discussion. The cell attached recordings (Supplementary Fig. 4) record ionic currents isolated from any changes in the extracellular space by the gigaseal of the patch pipette with the cell membrane. And yet these recordings still show large changes in spike amplitude. It is possible that these are caused by intracellular changes, such as changes in cell volume or intracellular Na^+^ concentration, but whole cell recordings, which minimize the latter, also show PDSs (*c.f.* examples in Trevelyan *et al.* 2006). Other animal work shows that spike shape changes also happen during far less intense network activity (Ranck, 1973; Steriade *et al.*, 1993; Harris *et al.*, 2000; Henze *et al.*, 2000). Henze *et al.* (2000)performed simultaneous intracellular and extracellular recordings of the same cell, to show correlated changes in action potential shape visible in both electrodes. Harris *et al.* (2000)analysed the reliability of spike sorting in tetrode recordings, noting that ‘error rates were increased by burst activity and during periods of cellular synchrony’. Overall, we conclude, on balance of evidence given the good correspondence on other measures between human and animal epileptic activity, that PDSs do indeed occur in the ictal core territory, but not in the penumbra.

Finally, we looked at the recovery of spike sorting after the seizure, and found that the spike shape and also the average firing rate across the population recovered to pre-seizure levels within a few hundred seconds. Our recovery data comes from three seizures analysed from a single patient, so should be viewed only as preliminary data, but what is clear is that the recovery of these two features is far in advance of the recovery of normal function in this subject, who experienced prolonged post-ictal lethargy with typical corresponding EEG changes (slowing and loss of faster frequencies). The protracted post-ictal state, which typically lasts from many minutes to hours or days (Rolak *et al.*, 1992; Gallmetzer *et al.*, 2004; Werhahn, 2010), therefore cannot be explained by an inability of neurons within the ictal core or penumbral territories to fire, but rather must be explained either by protracted altered function elsewhere in the brain, or alternatively in terms of persistently altered emergent features from the population activity in these areas. This is the topic of on-going investigation and will be covered in future work.

## Supplementary Material

Supplementary Fig. 2

## Abbreviations

MEA: microelectrode array
PDS: paroxysmal depolarizing shift
