# Supplementary material for: Single unit action potentials in humans and the effect of seizure activity
Source: Brain. 2015 Jul 17;138(10):2891–906. doi: 10.1093/brain/awv208 (PMC4671476; doi:10.1093/brain/awv208)
Supplement: Supplementary Fig. 2 [file brain_awv208_index.html]

Supplementary Data | Brain

## Supplementary Data

files

- Supplementary Data - zip file
