# Supplementary material for: Single unit action potentials in humans and the effect of seizure activity
Source: Brain. 2015 Jul 17;138(10):2891–906. doi: 10.1093/brain/awv208 (PMC4671476; doi:10.1093/brain/awv208)
Supplement: Supplementary Fig. 2 [file suppl_data.zip › brain-2014-02238-File010.pdf]

Title

Increased TRPC5 Glutathionylation Contributes to Striatal Neuron Loss in Huntington's Disease

Chansik Hong, Hyemyung Seo, Misun Kwak, Jeha Jeon, Jihoon Jang, Eui Man Jeong, Jongyun Myeong, Yu Jin Hwang, Kotdaji Ha, Min Jueng Kang, Kyu Pil Lee, Eugene C. Yi, In-Gyu Kim, Ju-Hong Jeon, Hoon Ryu, Insuk So

Supplementary Materials and Methods

Cell culture, transient transfection, plasmids and chemicals

HEK293 cells were maintained in Dulbecco's modified Eagle's medium (Hyclone, U.S.A.) supplemented with 10 % foetal bovine serum, 100 U/ml penicillin, and 100 μg/mL streptomycin according to the supplier's recommendations. The cells were seeded in 6- or 12-well plates depending on the experiment. For multiple cysteine mutation, single mutants of mTRPC5, provided by Mori (Yoshida, 2006) were generated by mutating he desired cysteine sites to serine in an overlapped manner by QuickChange site-directed mutagenesis (Agilent Technologies, U.S.A.). For transient transfection, the vector containing the GFP-tagged cDNA of hTRPC5 or mTRPC5 was used to transfect HEK293 cells cultured to 60-80 % confluence using FuGENE 6 (Roche Molecular Biochemicals, U.S.A.) for electrophysiological experiment or Lipofectamine 2000 (Invitrogen, U.S.A.) for molecular biology protocols according to the manufacturer's protocol. All experiments were performed 20-30 h after transfection. DTNB, 2-PDS, DTT, GSH, GSSG, BSO, H_2O_2, BCNU, NAC, and N-ethylmaleimide (NEM) were purchased from Sigma Aldrich (U.S.A.) except TCEP, which was obtained from Thermo Scientific (U.S.A.).

Electrophysiology

Patch pipettes were prepared from borosilicate glass and had resistances of 2-4 MΩ when filled with standard intracellular solutions. For whole-cell experiments, we used the standard pipette solution containing (in mM) 140 CsCl, 10 HEPES, 0.2 Tris-GTP, 0.5 EGTA, and 3 Mg-ATP. The pH of the pipette solution was adjusted to 7.3 using CsOH. Intracellular GSH or various concentrations of GSSG were adjusted by adding GSH or GSSG to the pipette solution. The external bath medium composition was as follows (in mM): 135 NaCl, 5 KCl, 2 $CaCl_2$, 1 $MgCl_2$, 10 glucose, and 10 *N*-[2-hydroxyethyl]piperazine-*N*'-[2-ethanesulfonic acid] (HEPES). The pH of the medium was adjusted to 7.4 using NaOH. Voltage ramp pulses were applied from +100 to -100 mV for 500 ms at -60 mV holding potential. The calculated junction potential between the pipette and bath solutions used for all cells was 5 mV (pipette negative), as determined by pClamp 10.2 software. No junction potential correction was applied. Experiments were performed at room temperature (18-22 °C). Cells were continuously perfused at a rate of 0.5 ml/min.

Intracellular Ca^{2+} measurement with Fura-2

The ratiometric measurements of $[Ca^{2+}]_i$ were obtained using Fura-2-AM (molecular probe, U.S.A.). The cells were grown in 24-well dishes and loaded with 5 μM Fura-2-AM for 30 min at 37 °C. Fura-2 fluorescence was measured at 510 nm emission with 340/380 nm dual excitation using a DG-4 illuminator. The experiments were performed in a normal solution containing (in mM) 145 NaCl, 3.6 KCl, 10 HEPES, 2 $CaCl_2$, 1 $MgCl_2$, and 5 glucose. The pH of the solution was adjusted to 7.4 using NaOH.

FRET-based Ca^{2+} measurement using Cameleon YC6.1

Three FRET images (cube settings for CFP, YFP, and Raw FRET) were obtained from a pE-1 Main Unit connected to 3 FRET cubes (excitation, dichroic mirror, filter) through a fixed

collimator: CFP (ET435/20m, ET CFP/YFP/mCherry beamsplitter, ET470/24m, Chroma); YFP (ET500/20m, ET CFP/YFP/mCherry beamsplitter, ET535/30m, Chroma); and Raw FRET (ET435/20m, ET CFP/YFP/mCherry beam-splitter, ET535/30m, Chroma). The excitation LED and filter were sequentially rotated, the rotation period for each of the filter cubes was ~0.5 s, and all images (three for CFP/YFP/Raw FRET, respectively) were obtained within 1.5 s. Each of the images was captured on a cooled 10 MHz (14 bit) CCD camera (DR-328G-C01-SIL: Clara, ANDOR technology, USA) with an exposure time of 100 ms with 2 × 2 binning (645 × 519 pixels) under the control of MetaMorph 7.6 software (Molecular Devices, Japan). To obtain the FRET efficiency of a cell, images were captured with an IX70 Olympus microscope equipped with a 60× oil objective, and the three-cube FRET method was used to calculate the efficiency (Erickson, 2001). Calcium mobilization was measured using Cameleon YC6.1 (generous gift from M. Ikura). Using a single excitation wavelength at 405 nm, which solely excites CFP, images and fluorescence emissions data for both CFP and YFP were collected. The experiments were performed in live neurons in the presence and absence of extracellular calcium, and the data obtained from each individual cell were used to calculate the ratios, which reflected the energy transferred. The background signal was subtracted from the values obtained after drug injection.

Western blot, co-immunoprecipitation (Co-IP), and surface biotinylation

In Co-IP experiments for the detection of glutathionylated protein, 500 μl of cell lysate was incubated with 0.2 mM NEM for 15 min at room temperature after treatment with 2 mM GSSG or 100 μM BCNU. As described by Chen et al. (2010), the samples were incubated with 1 μg of IP antibody and 30 μl of protein G-agarose beads at 4 °C overnight with gentle rotation. The beads were subsequently washed three times with wash buffer (0.1 % Triton X-100, 50 mM Tris-Cl, 150 mM NaCl, 1 mM EDTA, pH 7.5), and the precipitates were eluted with 30 μl of 2× Laemmli buffer and subjected to western blot analysis. To eliminate background noise due to non-specific immunoglobulins (Igs) in some IP and immunoblotting using tissue samples, we used control Igs and specific secondary TrueBlot antibodies (Rockland, U.S.A.).

For surface biotinylation, PBS-washed cells were incubated in 0.5 mg/ml sulfo-NHS-LC-biotin (Pierce, U.S.A.) in PBS for 30 min on ice. The biotin was then quenched by the addition of 100 mM glycine in PBS. The cells were then processed as described above to prepare cell extracts. A 40-μl aliquot of 1:1 slurry of immobilized avidin beads (Pierce, U.S.A.) was added to 300 μl of cell lysate (500 μg protein). After incubation for 1 h at room temperature, the beads were washed three times with 0.5 % Triton-X-100 in PBS, and proteins were extracted in sample buffer. The collected proteins were then analysed by western blot.

Reverse transcription polymerase chain reaction (RT-PCR) analysis

Total RNA was extracted from Q7 or Q111 cells using TRIzol reagent (Invitrogen, U.S.A.). Reverse transcription was performed using a commercial kit according to the manufacturer's instruction (Invitrogen, U.S.A.). RT-PCR of TRPC5 mRNA was performed using TRPC5 forward primer (TAG TAC TAC TGG CTT TTG CCA ACG), and TRPC5 reverse primer (ATT CAG CAG CAC TAC CAG GGA GAT). The following conditions were used for PCR: pre-heating (94 °C, 2 min); 35 cycles of denaturation (94 °C, 30 sec), annealing (50 °C, 30 sec), and extension (72 °C, 60 sec); and a final extension (72 °C, 10 min).

Quantitative real-time polymerase chain reaction (qRT-PCR) analysis

Total RNA was isolated from cells using a commercial extraction system (Macherey-Nagel, Germany). A total of 1 μg of RNA was used to prepare using the First Strands cDNA Synthesis Kit (TOYOBO, Japan) according to the manufacturer's protocols. cDNA was

amplified from each sample by qRT-PCR using SYBR Green Supermix (TOYOBO, Japan). The qRT-PCR cycling conditions were as follows: denaturation for 3 min at 95 °C; 40 cycles of amplification for 15 s at 95 °C, 15 s at 60 °C, 20 s at 70 °C; and 30 s at 72 °C. RNA quantities were normalized using GAPDH mRNA as a reference (Jeon, 2012a). The sequences of the qRT-PCR primers for mouse samples were as follows: mouseTRPC1 forward primer (TCG TGG GCC GCG ATG ATG GC), mouse TRPC1 reverse primer (CAG CCT TGT CGC ACG CCA GC), mouse TRPC5 forward primer (CCC ACA GAA GTA TCA TGA CCT), and mouse TRPC5 reverse primer (TTC TGG GTG AGA TCA GAT AGG).The sequences of the qRT-PCR primers for human samples were as follows: human TRPC1 forward primer (TAT CTC TAC CCA AGC CCC AT), humanTRPC1 reverse primer (GAA TTC CAC CTC CAC AAG AC), human TRPC5 forward primer (TTA TGC TTC TCC TGG CTT CTC), and human TRPC5 reverse primer (CAG GGA AAT AGT TGC CAG GTA).

Cellular glutathione assay

Q7 and Q111 cells were seeded at 85 % confluency in white, clear-bottom 96-well plates (Corning Incorporated, U.S.A.). After treatment with 100 µM BSO for 24 h or 100 µM BCNU for 6 h, the cells were washed with PBS twice and stored at -80 °C until the glutathione assay. Total GSH and GSSG concentrations were measured using the GSH/GSSG-Glo Assay kit (Promega, U.S.A.). The reduced form (GSH) concentration was calculated by subtracting the oxidized form (GSSG) concentration from the total glutathione concentration.

Gene silencing using siRNA (siTRPC1 and siTRPC5)

For gene silencing, siRNA was transfected using the RNAi Max Transfection Reagent (Invitrogen, U.S.A.) according to the manufacturer's instructions. Mouse TRPC5 siRNA (Cat.1441728) and mouse TRPC1 siRNA (Cat.1441678) were purchased from Bioneer (South Korea). Control siRNA (Cat.D-001210-01-05) was purchased from Dharmacon (U.S.A.). The silencing effects were evaluated using RT-PCR or western blotting.

Animal care and experimental design

YAC128 mutant HD transgenic mice were purchased from Jackson Laboratory (FVB-Tg(YAC128)53Hay/J, Jackson Laboratory, U.S.A.). Mice were housed under a 12-h light/dark cycle, in a room with controlled temperature (25 ± 1 ºC) and free access to food and water. For adaptation, mice were acclimated to laboratory conditions for at least 1 h before all behaviour tests. In total, 18 female transgenic mice and 16 female littermate control mice (12-15 months old) were used in these experiments. All procedures were approved by the Hanyang Institutional Animal Care and Use Committee (HY-IACUC-09-017) and performed according to their guidance. Mice were intraperitoneally injected with 0.5 mg/kg ML204 or vehicle (0.4 % DMSO with saline) daily for 8 days. The mice were evaluated in the olfactory sensitivity test and tail suspension test on the 6th day and the rotarod test and open-field test on the 7th day. On the 8th day, all mice were sacrificed 2 hours after the final injection. Mice were dissected to obtain several brain regions, including the striatum (ST) and frontal cortex (FC), for biochemical analysis or were perfused for immunohistochemical analysis.

GSH/GSSG assay

Dissected brain tissues were fractionized as described previously (Seo, 2008). Dissected brain tissues were homogenized in 100 µl of HB buffer (50 mM Tris (pH 8.0), 150 mM NaCl, 5 mM EDTA, 1 % Triton X-100, protease and phosphatase inhibitors; 10 µg/ml aprotinin, 25 µg/ml leupeptin, 10 µg/ml of pepstatin, 10 µg/ml phenylmethanesulfonyl fluoride, 50 mM sodium fluoride and sodium orthovanadate), then centrifuged at 1,400 ×*g* for 5 minutes at 4 °C. After centrifugation, the supernatant was transferred to new tube and then centrifuged at

7,900 ×*g* for 15 min at 4 °C. The supernatant was used as the cytosolic fraction. To detect GSH/GSSG levels in the cytosolic fractions of the frontal cortex and striatum, we performed GSH/GSSG ratio detection assay (Abcam, U.S.A.). We used a Thiol Green Indicator Reaction Mixture, GSH standards, GSSG standards. Test samples (20 μg) were loaded into an assay plate (96-well plate) with the Thiol Green Indicator Reaction Mixture. After incubation at room temperature for 15 min, enzyme activity was detected at 490/520 nm and normalized for protein concentration before statistical analysis.

Immunohistochemistry (IHC)

Mice were perfused with 0.1 M phosphate-buffered saline (PBS), followed by 4 % paraformaldehyde (PFA). The brains were then extracted and additionally post-fixed with 4 % PFA for 3 h at 4 °C. After post-fixing, the brains were equilibrated in 20 % sucrose (in 0.1 M PBS) for 2 days at 4 °C. After the equilibration, brains were stored in 0.03 % sodium azide (in 0.1 M PBS), then sectioned into 30-μm thicknesses using a freezing microtome (Thermo Scientific, U.S.A.) and preserved in 0.03 % sodium azide (in 0.1 M PBS) at 4 °C until use. We immunostained the BLA, DRN, striatum (ST) and SNpc regions of the brain. Brain sections were incubated in 0.3 % H_2O_2 for 30 min to remove endogenous peroxidase, followed by three washes in DPBS for 10 min. After washing, sections were incubated in 10 % NGS DPBS for 1 h for blocking and then incubated for 3 h at room temperature with anti-cholinacetyltransferase (ChAT) (1:500, Millipore, U.S.A.), anti-tryptophan hydroxylase 2 (TPH2) (1:200, Novus Biologicals, U.S.A.), anti-dopamine- and cAMP-regulated neuronal phosphoprotein (DARPP32) (1:500, Abcam, U.K.) or anti-tyrosine hydroxylase (TH) (1:500, Pel-Freez U.S.A.), anti-TRPC5 (1:500, NeuroMab, U.S.A.) or anti-TRPC1 (1:500, NeuroMab, U.S.A.). After primary antibody reaction, sections were washed 3 times in DPBS then incubated for 1 hour at room temperature with a horseradish peroxidase (HRP)-conjugated polyclonal anti-rabbit antibody (1:500, Vector, U.S.A.) or HRP-conjugated polyclonal anti-rabbit antibody (1:500, Vector) as the secondary antibody. ChAT-positive cells, TPH2-positive cells, DARPP32-positive cells and TH-positive cells were visualized with a DAB substrate kit (Vector, U.S.A.). Sections were mounted on 0.3 % gelatinine-coated cover glasses and air-dried. The cover glasses were mounted in Permount medium. Images of ChAT-immunoreactive cells in the BLA, TPH2-immunoreactive cells in the DRN, DARPP32-immunoreactive cells in the ST and TH-immunoreactive cells in the SNpc were obtained using a Carl Zeiss microscope (Axio Observer, Germany). Analysis was performed by two different assessors in a blinded manner using the Image J program (version 1.46r; NIH).

Behaviour assessment

Rotarod test The motor balance of the mice on rotarod (Panlab, Spain) was evaluated as described in a previous report with modifications (Seo, 2008). Mice were subjected to the rotarod test in 4 trials in 1 day. The speed of the rotarod was fixed at 5 rpm, and each trial was performed for a period of 60 seconds. The latency to fall was recorded for each mouse in each trial.

Open-field test Mice were submitted to the open-field test to assess anxiety and movement. Mice were placed in the centre of a clear acrylic box (27.5 × 27.5 cm) and allowed to move freely. The bottom surface of the box was divided into 25 squares (5.5 × 5.5 cm each) with black lines. Each trial was 4 min, and mice were subjected to 3 consecutive trials. The movements (line cross, rearing, wall rearing, and grooming) of the mice were counted and recorded for each mouse in each trial. A line cross was defined as a movement in which both hind legs completely crossed a line. Rearing was counted as a movement in which the mouse stood on its hind leg and tail, put both forelegs to the wall, and leaned against the wall.

Olfactory sensitivity test In this test, an adapted black Y-maze (30 × 5 × 12 cm) with 2 arms was used. Each arm contained a plastic dish (with a 3 cm diameter) on which soaked paper towels were placed. The paper towels were soaked with 30 μl of 100 % butanol (odorized compartment), an odorant that is repulsive to mice (Deiss, 1997), or 200 μl of distilled water (control compartment). The odorized compartment and control compartment were randomly distributed to either the right or left arm in each test. After each test, the Y-maze was carefully washed with 70 % ethanol and distilled water to remove remaining odour. Mice were placed separately at the end of the start arm and then allowed to move freely for 5 min. The movements of mice were video recorded and analysed with a SMART video tracking system (Panlab, Spain). The time spent in each arm by each mouse was then recorded.

Tail suspension test Mice were tested for depression-like activity using the tail suspension test modified from a previous report (Steru, 1985). Mice were individually suspended by their tails from a hook at a height of 35 cm using a clothespin to hold 2 cm of the tail on the hook. The movements of the mice were observed, and video was recorded for 6 min. The time of immobility as a measure of depression-like activity corresponded to the time a mouse was completely motionless.

Supplemental Table and Figure Legends

Table S1. Alignment of the full sequences on mouse TRPC1, TRPC4, and TRPC5 channels.

Figure S1 - TRPC5 activation by compounds increasing oxidative stress by multiple pathways and reversal but reductants.

A. TRPC5 activation by treatment with DTNP (30 μM).

B. TRPC5 activation by treatment with 2-PDS (100 μM).

C. TRPC5 activation by internally infused DTNB (200 μM).

D. Lack of TRPC5 activation by extracellular DTNB (1 mM).

E and F. Reversal of DTNP-activated TRPC5 current by the reducing agent DTT. Cells were treated with DTNP (30 μM) to activate TRPC5 (red bar traces), which was reversed by 10 mM DTT (**E**, green traces), but not by the cell-impermeable agent 1 mM TCEP (**F**, blue traces).

Figure S2 - Reciprocal regulation of TRPM2 by cellular redox potential.

A. The assumed redox sensor hTRPM2 is activated by pipette 300 μM cADPR, but is not activated by 5 mM GSSG (red), or 30 μM DTNP (blue).

Figure S3 - Role of cysteine mutants on direct activation of TRPC5 by GTPγS and DTNP.

A. TRPC5 current of the indicated cysteine mutants was activated by infusing the cells

with GTPγS (0.2 mM).

B. Effect of single and double cysteine mutations on TRPC5 activation by DTNP (30 μM).

C. The C176S-C178S double cysteine mutant does not affect TRPC5 surface expression assayed by biotinylation.

D. A typical doubly-rectifying *I-V* current of WT and the double mutant activated by 200 μM La^{3+}.

E. The C176S-C178S double cysteine mutant inhibits the TRPC5-mediated Ca^{2+} influx activated by H_2O_2 (WT; $N = 33$, C176S-C178S; $N = 30$). Ca^{2+} was measured in HEK293 cells transfected with TRPC5 and loaded with Fura-2.

Figure S4 - Activation of TRPC5 in mutant huntingtin striatal cells.

All experiments were performed in Q7 and Q111 cells, unless otherwise stated.

A. The presence of TRPC5 was detected by a polyclonal TRPC5 antibody (Alomone labs) using a blocking peptide as a negative control.

B. Activation of endogenous TRPC5-like current in Q7 by a $G\alpha_{i3}$ constitutively active $G\alpha_{i3}$QL mutant that activates TRPC5 (Jeon, 2012b).

C. Activation of endogenous TRPC5-like current in Q111 cell by $G\alpha_{i3}$QL.

D. TRPC5 current activated by GSSG (5 mM) is inhibited by ML204 (10 μM).

E. Efficiency of knockdown of TRPC5 mRNA by siRNA in Q7 and Q111 cells as assayed by RT-PCR.

Figure S5 - The effect of TRPC5 blocker in neuropathology of YAC128 HD mice.

All experiments were performed with littermate control (WT) and YAC128 HD mice (YAC128). * denotes $p < 0.05$ and n.s., not significant. Scale bar indicates 100 μm.

A. The presence of TRPC5 was detected by a polyclonal TRPC5 antibody (Alomone labs) using over-expressed TRPC5 lysates and a blocking peptide in the striatum of WT and YAC128 mice.

B-G. Representative IHC image for anti-ChAT in the BLA (A), anti-TPH2 in the DRN (**C**) or anti-TH in the SNpc (**E**) of YAC128 mice on neuronal cell population after ML204 administration.

B. Cholinergic neurons in the BLA (**A**) decreased in YAC128 mice compared to WT.

C. The number of cholinergic neuronal cells increased in the BLA region of YAC128 mice compared to WT upon ML204 administration.

D. Serotonergic neurons in the DRN did not show a significant change in YAC128 mice compared to WT upon ML204 administration.

E. The number of TPH2-positive serotonergic cells was not significantly changed in the DRN of YAC128 mice upon ML204 administration.

F. Dopaminergic neurons in the SNpc did not show a significant change in YAC128 mice compared to WT upon ML204 administration.

G. The number of TH-positive dopaminergic cells was not significantly changed in the SNpc of YAC128 mice upon ML204 administration.

H-J. The effects of ML204 on the motor or non-motor behaviour of YAC128 mice: the open-field test (**H**), the odorant avoidance test (**I**), and the tail suspension test (**J**):

H. ML204 did not affect the grooming behaviour of WT and YAC128 mice in the open-field test (WT; $N = 10$, YAC; $N = 10$).

I. ML204 did not affect the olfactory sensitivity of both mice in the olfactory sensitivity test (WT; $N = 10$, YAC; $N = 10$).

J. ML204 did not affect the spasticity of both mice in the tail suspension test (WT; $N = 10$, YAC; $N = 10$, $p = 0.1$).

Figure S6 - S-glutathionylation of TRPC5 in mice and human striatum of HD.

The experiments were performed with HD transgenic mice (YAC128 and R6/2) and human HD patients. * denotes $p < 0.05$ and n.s., not significant.

A. mRNA levels of TRPC1 or TRPC5 in the human HD patients (control; $N = 5$, HD patients; $N = 5$).

B. The presence of TRPC5 was detected by a polyclonal TRPC5 antibody (Alomone labs) using over-expressed pcDNA or mTRPC5 lysates as a positive control in the human striatum of normal and HD patients.

C. The endogenous TRPC5 is S-glutathionylated in the striatum tissue of R6/2 HD transgenic mice.

D. The endogenous TRPC5 is S-glutathionylated in the the striatum tissue of HD animals compared to the controls. The IP and input blots with the dotted line using anti-GSH were performed under non-reducing condition. A rabbit polyclonal TRPC5 antibody (Alomone labs) was used for immunoprecipitation.

E.
