# Supplementary material for: Single unit action potentials in humans and the effect of seizure activity
Source: Brain. 2015 Jul 17;138(10):2891–906. doi: 10.1093/brain/awv208 (PMC4671476; doi:10.1093/brain/awv208)
Supplement: Supplementary Fig. 2 [file suppl_data.zip › brain-2014-02238-File011.pdf]

Supplemental Table 1

The homology of TRPC5(Mouse), TRPC4(Mouse), and TRPC1(Mouse)

```
                                                                                   65
TRPC5(Mouse)   MAQLYYKKVNYSPYRDRIPLQIVRAETELSAEEKAFLSAVEKGDYATVKQALQEAEIYYNVNINCMDPLGRSALLIAIEN 080
TRPC4(Mouse)   MAQFYYKRNVNAPYRDRIPLRIVRAESELSPSEKAYLNAVEKGDYASVKKSLEEAEIYFKININCIDPLGRTALLIAIEN 080
TRPC1(Mouse)   ---L--------P----V-------E------EK-FL-A-DKGDY--VKK-LEE-------NINC-D-LGR-A--I-IEN 110

TRPC5(Mouse)   ENLEIMELLLNHSVYVGDALLYAIRKEVVGAVELLLSYRKPSGEKQVPTLMMDTQFSEFTPDITPIMLAAHTNNYEIIKL 160
TRPC4(Mouse)   ENLELIELLLSFNVYVGDALLHAIRKEVVGAVELLLNHKKPSGEKQVPPILLDKQFSEFTPDITPIILAAHTNNYEIIKL 160
TRPC1(Mouse)   E-LDI-QLLL-------DALL-AI--EVVGAVD-LLNHRK-S-----I--LM---QY-E-T-DV-PVILAAH-NNYEI--M 191
                                                   TRPC1β isoform
                               176 178 181
TRPC5(Mouse)   LVQKRVTIPRPHQIRCNCVECVSSSEVDSLRHSRSRLNIYKALASPSLIALSSEDPILTAFRLGWELKELSKVENEFKAE 240
TRPC4(Mouse)   LVQKGVSVPRPHEVRCNCVECVSSSDVDSLRHSRSRLNIYKALASPSLIALSSEDPFLTAFQLSWELQELSKVENEFKSE 240
TRPC1(Mouse)   L----VS-P-PH-V-CEC--C------DSLRHSR-RLDIY--LASP-LI-L--EDPIL-AF-LS-DLKELS-VE-EF--D 273

                     248                                                         307
TRPC5(Mouse)   YEELSQCKLFAKDLLDQARSSRELEIILNHRDDHSEELDPQKYHDLAKLKVAIKYHQKEFVAQPNCQQLLATLWYDGFP 320
TRPC4(Mouse)   YEELSRQCKQFAKDLLDQTRSSRELEIILNYRDDN-SLIEEQSGNDLARLKLAIKYRQKEFVAQPNCQQLLASRWYDEFP 319
TRPC1(Mouse)   YEEL-RQCK-FAKDLL-Q-R-SRELEVILNH----------E---NL-RLKLAIKY-QKEFVSQSNCQQ-L-T-WF---- 356

TRPC5(Mouse)   GWRRKHWVVKLLTCMTIGFLFPMLSIAYLISPRSNLGLFIKKPFIKFICHTASYLTFLFMLLLASQHIVRTDLHVQGPPP 400
TRPC4(Mouse)   GWRRRHWAVKMVTCFIIGLLFPVFSVCYLIAPKSPLGLFIRKPFIKFICHTASYLTFLFLLLLASQHIDRSDLNRQGPPP 399
TRPC1(Mouse)   G-RRK----K-T----VG---PVLS-CYLIAPKS--G--I--PF-KFI-H-ASY-TFL-LL-L-S--------N--GP-- 436

TRPC5(Mouse)   TVVEWMILPWVLGFIWGEIKEMWDGGFTEYIHDWWNLMDFAMNSLYLATISLKIVAYVKYNGSRPREEWEMWHPTLIAEA 480
TRPC4(Mouse)   TIVEWMILPWVLGFIWGEIKQMWDGGLQDYIHDWWNLMDFVMNSLYLATISLKIVAFVKYSALNPRESWDMWHPTLVAEA 479
TRPC1(Mouse)   --ID-L---WI-G-IW-DIK-LW--GL-DF--E--N-L-FVMNSLYLAT--LKVVA—KF------R--WD--HPTLVAE- 516

                                                                                553  558
TRPC5(Mouse)   LFAISNILSSLRLISLFTANSHLGPLQISLGRMLLDILKFLFIYCLVLLAFANGLNQLYFYYETRAIDEPNNCKGIRCEK 560
TRPC4(Mouse)   LFAIANIFSSLRLISLFTANSHLGPLQISLGRMLLDILKFLFIYCLVLLAFANGLNQLYFYYEETKG---LSCKGIRCEK 556
TRPC1(Mouse)   LFA--NVLS-LRL--MYT--S-LGPLQISMG-ML-D--KFL--F-LVL--F--GL-QLY----T----E---C-GI-CE- 594

TRPC5(Mouse)   QNNAFSTLFETLQSLFWSVFGLLNLYVTNVKARHEFTEFVGATMFGTYNVISLVVLLNMLIAMMNNSYQLIADHADIEWK 640
TRPC4(Mouse)   QNNAFSTLFETLQSLFWSIFGLINLYVTNVKAQHEFTEFVGATMFGTYNVISLVVLLNMLIAMMNNSYQLIADHADIEWK 636
TRPC1(Mouse)   Q-N-F-----T---LFW-IF-L---FVT------E---FVGA---GTYNVV--IVL--LLVAML--SFQLIANH-D-EWK 677

                                                          682
TRPC5(Mouse)   FARTKLWMSYFDEGGTLPPPFNIIPSPKSFLYLGNWFNNTFCPKRDPDGRRRRHNLRSFTERHADSLIQNQHYQEVIRNL 720
TRPC4(Mouse)   FARTKLWMSYFEEGGTLPTPFNVIPSPKSLWYLVKWIWTHLCKKK---MRRKPESFGTIGRRAADNLRRHHQYQEVMRNL 713
TRPC1(Mouse)   FAR-KLWLSYFDD--TLPPPFNIIPSPK---YM--------C---------K-----S—E--------R---YQ-VM--L 738

TRPC5(Mouse)   VKRYVAAMIRNSKTHEGLTEENFKELKQDISSFRYEVLDLLGNRKHPRRSLSTSSADFSQRDDTNDGSGGARAKSKSVSF 800
TRPC4(Mouse)   VKRYVAAMIREAKTEEGLTEENVKELKQDISSFRFEVLGLLRGSKLSTIQSANAASSADSDEKSQSEGNGKDKRKNLSLF 793
TRPC1(Mouse)   V-RY---M-------D--T-EN--EL-QD-S-FR-EI-DLL-------------- 809
                                                                     TRPC4β isoform

TRPC5(Mouse)   NVG--CKKKACHGAPLIRTVPRASGAQGKPKSESSSKRSFMGPSFKKLGLFFSKFNGQTSEPTSEPMYTISDGIAQQHCM 878
TRPC4(Mouse)   DLTTLIHPRSAAIASERHNLSNGSALVVQEPPREKQRKVNFVADIKNFGLFHRRSKQNAAEQNANQIFSVSEEITRQQAA 873
TRPC1(Mouse)

TRPC5(Mouse)   WQDIRYSQME-KGKAEACSQSEMNLGE----VELGEVRGAAARSSECPLACSSSLHCASGICSSNSKLLDSSEDVFETWG 953
TRPC4(Mouse)   GALERNIELESKGLASRGDRSIPGLNEQCVLVDHRERNTDTLGLQVGKRVCSTFKSEKVVVEDTVPIIPKEKHAHEEDSS 953
TRPC1(Mouse)

TRPC5(Mouse)   EACDLLMHKWGDGQEEQVTTRL 975
TRPC4(Mouse)   IDYDLSPTDTAAH-EDYVTTRL 974
TRPC1(Mouse)
```
