# Supplementary material for: Single unit action potentials in humans and the effect of seizure activity
Source: Brain. 2015 Jul 17;138(10):2891–906. doi: 10.1093/brain/awv208 (PMC4671476; doi:10.1093/brain/awv208)

Supplemental Fig. 1

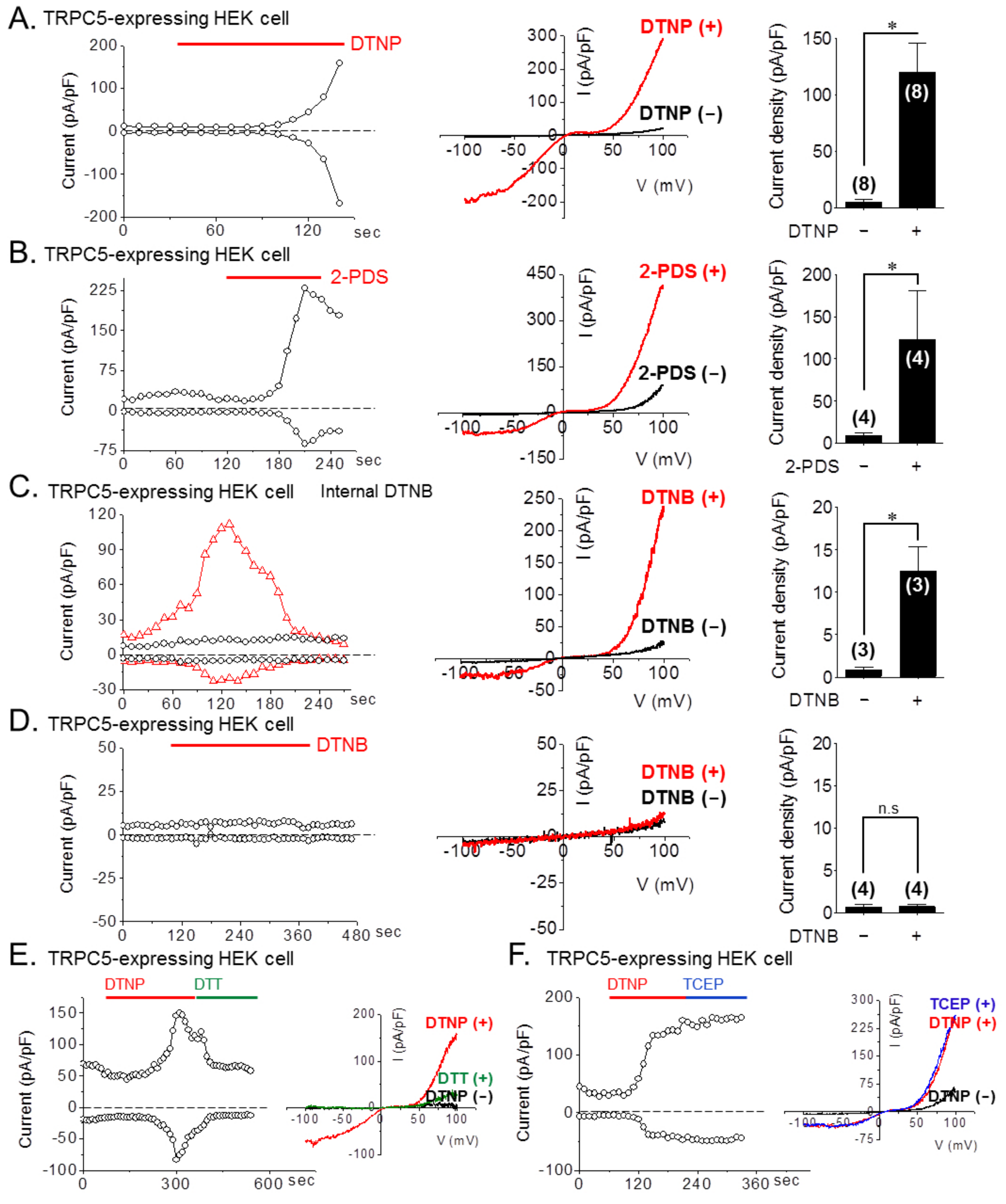

Supplement: Supplementary Fig. 2 [file suppl_data.zip › brain-2014-02238-File012.pdf]
