# Supplementary figures and images for: Single unit action potentials in humans and the effect of seizure activity
Source: Brain. 2015 Jul 17;138(10):2891–906. doi: 10.1093/brain/awv208 (PMC4671476; doi:10.1093/brain/awv208)

# Supplemental Fig. 4

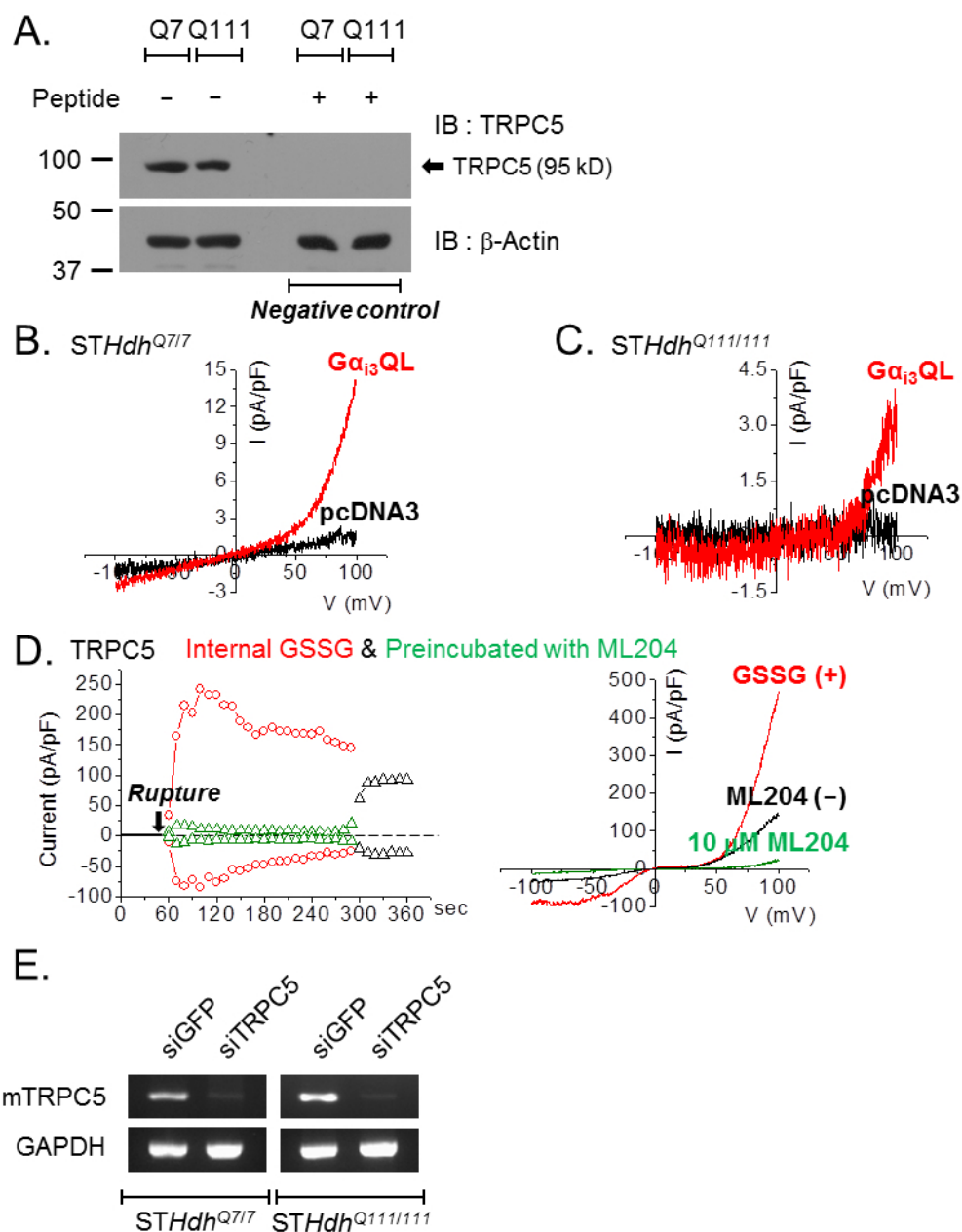

Supplement: Supplementary Fig. 2 [file suppl_data.zip › brain-2014-02238-File015.pdf]

# Supplemental Fig. 5

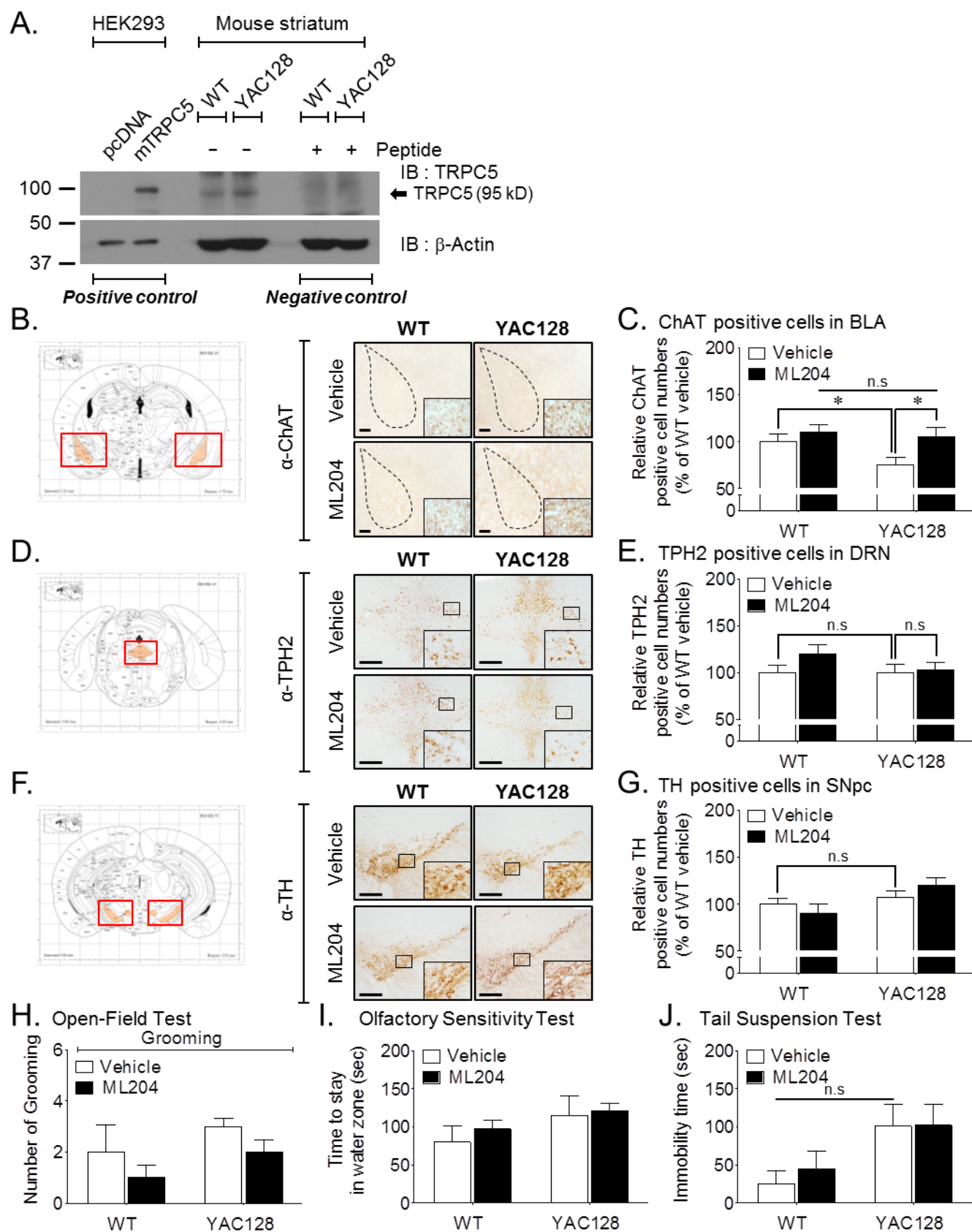

Supplement: Supplementary Fig. 2 [file suppl_data.zip › brain-2014-02238-File016.pdf]

# Supplemental Fig. 6

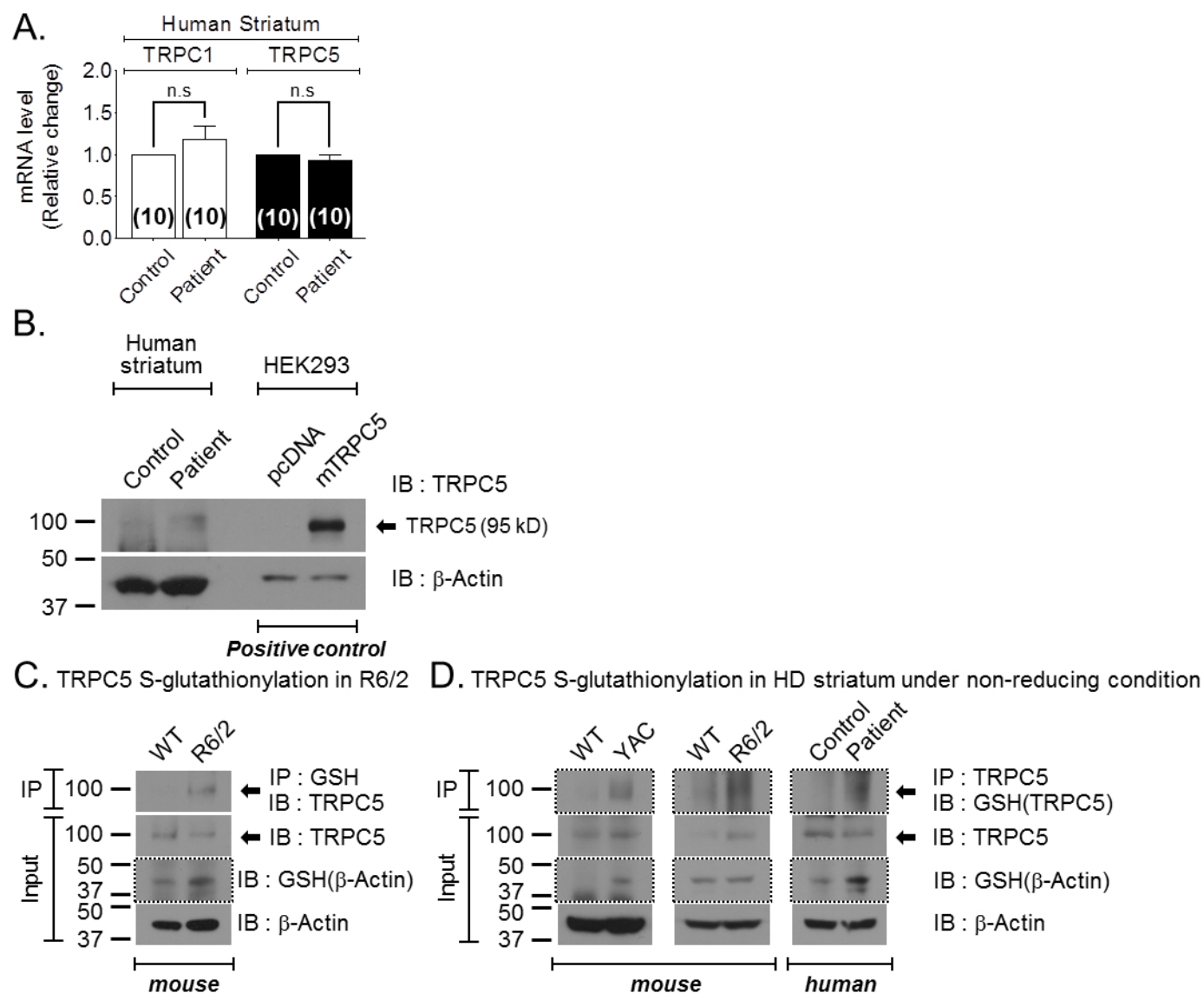

Supplement: Supplementary Fig. 2 [file suppl_data.zip › brain-2014-02238-File017.pdf]

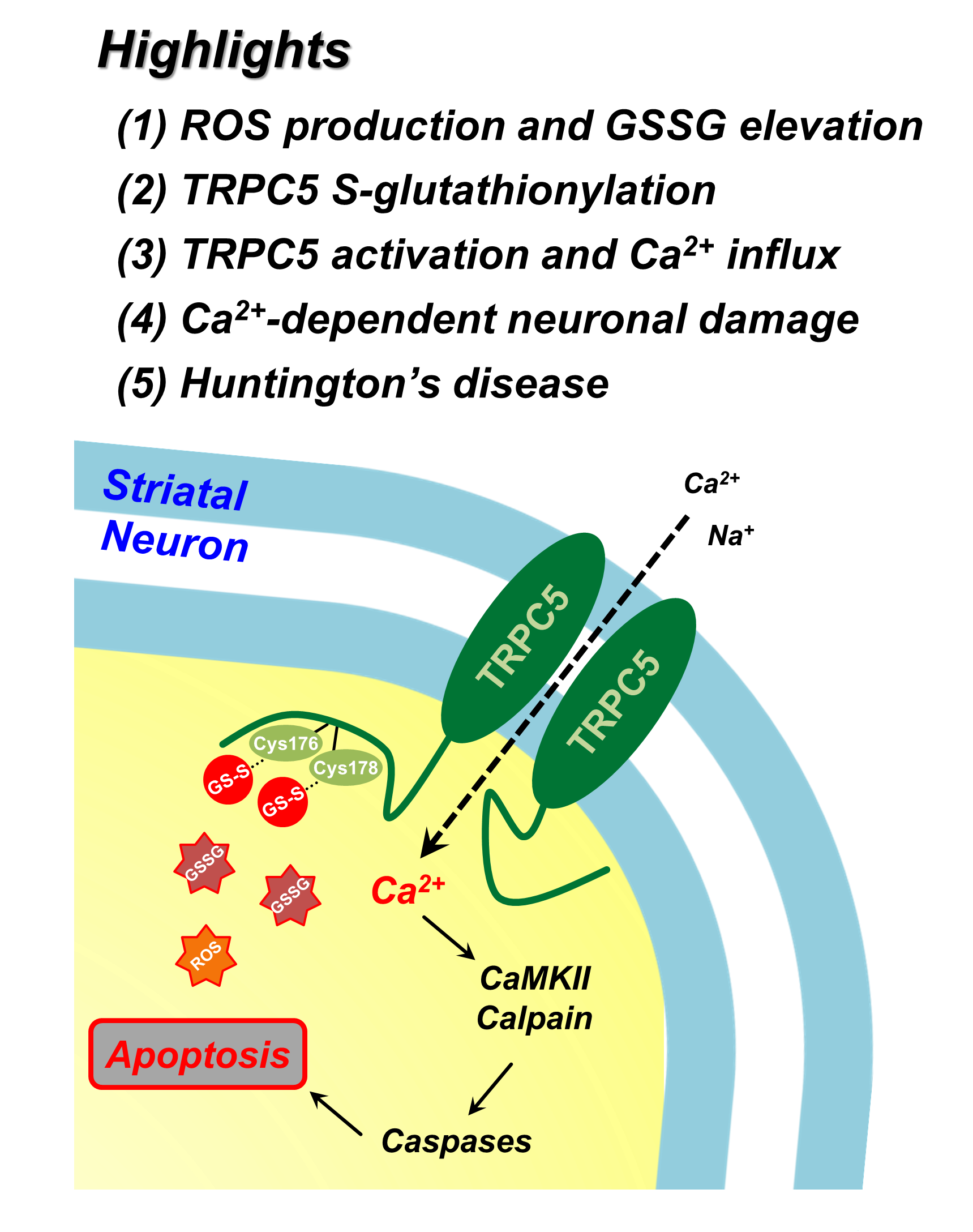

Supplement: Supplementary Fig. 2 [file suppl_data.zip › brain-2014-02238-File018.tif]

## Supplemental Fig. 2

A. hTRPM2-expressing HEK cell

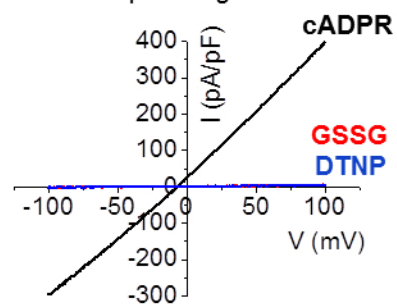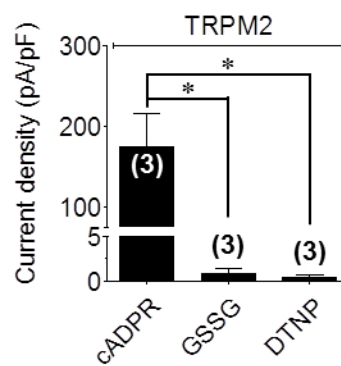

Supplement: Supplementary Fig. 2 [file suppl_data.zip › brain-2014-02238-File013.pdf]

# Supplemental Fig. 3

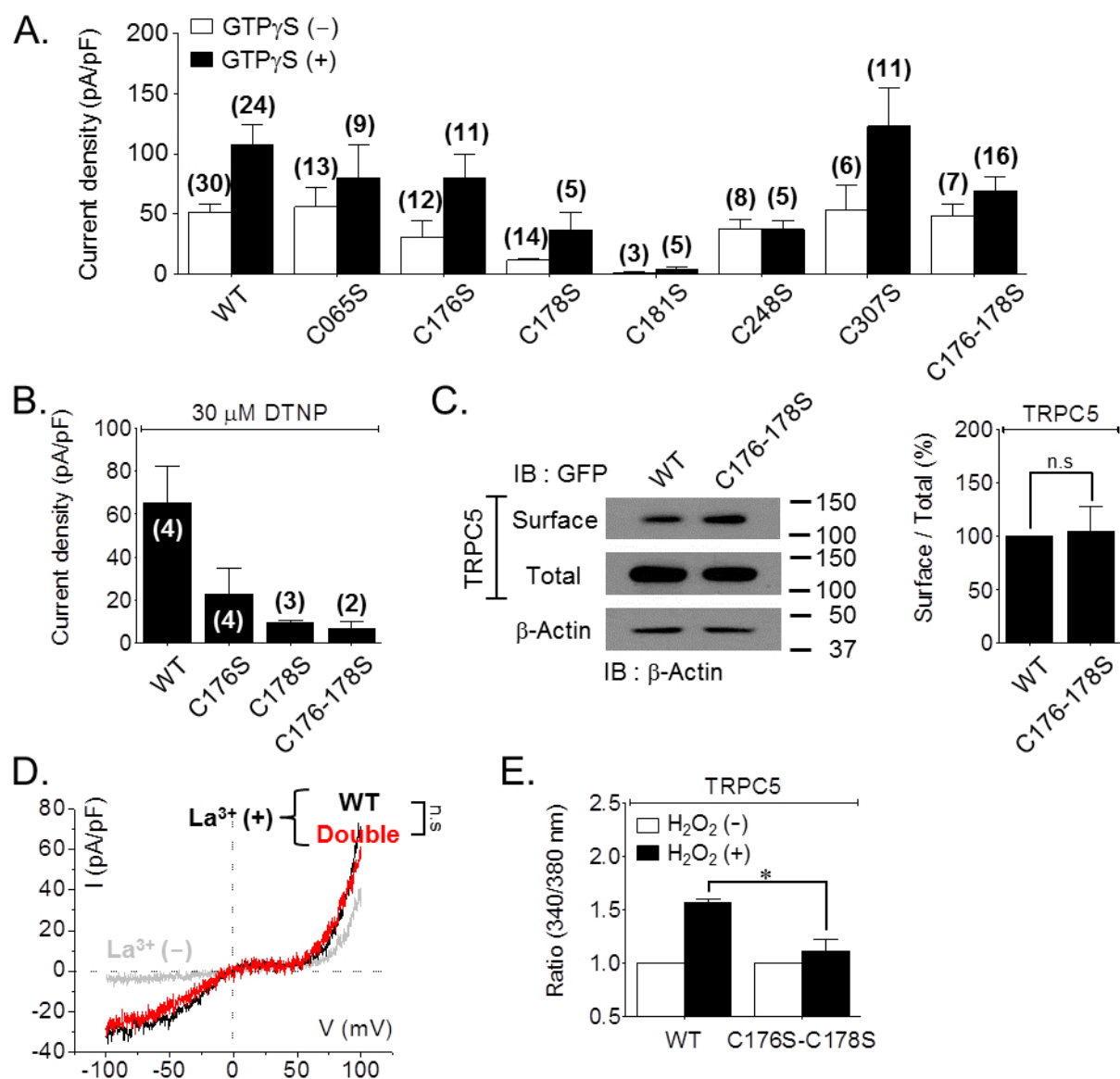

Supplement: Supplementary Fig. 2 [file suppl_data.zip › brain-2014-02238-File014.pdf]
